# Correction: Pooled prevalence and associated factors of traditional uvulectom among children in Africa: A systematic review and meta-analysis

**DOI:** 10.1371/journal.pone.0329891

**Published:** 2025-08-05

**Authors:** Solomon Demis Kebede, Kindu Agmas, Demewoz Kefale, Amare Kassaw, Tigabu Munye Aytenew

The first author’s initials appear incorrectly in the citation. The correct citation is: Kebede SD, Agmas K, Kefale D, Kassaw A, Aytenew TM (2025) Pooled prevalence and associated factors of traditional uvulectomy among children in Africa: A systematic review and meta-analysis. PLoS ONE 20 [1]: e0316755. https://doi.org/10.1371/journal.pone.0316755.

The word “uvulectomy” is misspelled in the article title. The correct title is: Pooled prevalence and associated factors of traditional uvulectomy among children in Africa: A systematic review and meta-analysis. The correct citation is: Kebede SD, Agmas K, Kefale D, Kassaw A, Aytenew TM (2025) Pooled prevalence and associated factors of traditional uvulectomy among children in Africa: A systematic review and meta-analysis. PLoS ONE 20 [1]: e0316755. https://doi.org/10.1371/journal.pone.0316755

## References

[1] Demis Kebede S, Agmas K, Kefale D, Kassaw A, Aytenew TM. Pooled prevalence and associated factors of traditional uvulectom among children in Africa: A systematic review and meta-analysis. PLoS One. 2025;20(1):e0316755. doi: 10.1371/journal.pone.0316755 39874381 PMC11774377

