# Supplementary material for: Pooled prevalence and associated factors of traditional uvulectom among children in Africa: A systematic review and meta-analysis
Source: PLoS One. 2025 Jan 28;20(1):e0316755. doi: 10.1371/journal.pone.0316755 (PMC11774377; doi:10.1371/journal.pone.0316755)
Supplement: S2 File 2 — (DOCX) [file pone.0316755.s002.docx]

Supplementary file 2: Quality Assessment of the Included Studies Using JBI Quality Appraisal Criteria

|  | Criteria | | | | | | | | | Score | Quality |
| --- | --- | --- | --- | --- | --- | --- | --- | --- | --- | --- | --- |
| Author | Was The Sample Frame Appropriate? | Was Sampling Appropriate? | Was The Sample Size Adequate? | Were The Study Subjects And The Setting Described In Detail? | Was The Data Analysis Conducted With Sufficient Coverage Of The Identified Sample? | Were Valid Methods Used For The Identification Of The Condition? | Was The Condition Measured In A Standard, Reliable Way For All Participants? | Was There Appropriate Statistical Analysis? | Was The Response Rate Adequate, And If Not, Was The Low Response Rate Managed Appropriately? |  |  |
| Adoga, (2011) | yes | yes | yes | yes | yes | yes | no | yes | no | 6 | Low risk |
| Bayih Et Al., (2020) | yes | yes | yes | no | yes | yes | no | yes | yes | 7 | Low risk |
| Djakounda, (1994) | yes | yes | yes | yes | yes | yes | yes | yes | yes | 9 |  |
| Farouk Et Al., (2023) | Yes | Yes | Yes | Yes | Yes | Yes | Yes | Yes | Yes | 9 | Low risk |
| Gebrekirstos Et Al., (2013) | yes | yes | yes | not clear | yes | yes | yes | yes | yes | 8 | Low risk |
| Gebrekrstos Et Al., (2014) | yes | yes | yes | yes | no | yes | yes | no | no | 6 | Low risk |
| Kebede Et Al., (2017) | yes | yes | no | yes | no | yes | yes | yes | no | 6 | Low risk |
| Kefelew Et Al., (2023) | yes | yes | yes | yes | yes | yes | yes | yes | yes | 9 | Low risk |
| Mike, (2010) | yes | yes | yes | yes | yes | yes | yes | yes | yes | 9 | Low risk |
| Oluwatosin Et Al., (2016) | yes | yes | no | yes | yes | no | no | yes | yes | 6 | Low risk |
| Yirdaw Et Al (2022) | yes | yes | yes | yes | not clear | yes | yes | yes | yes | 8 | Low risk |
