# Supplementary material for: Pooled prevalence and associated factors of traditional uvulectom among children in Africa: A systematic review and meta-analysis
Source: PLoS One. 2025 Jan 28;20(1):e0316755. doi: 10.1371/journal.pone.0316755 (PMC11774377; doi:10.1371/journal.pone.0316755)
Supplement: S4 File 4 — (DOCX) [file pone.0316755.s004.docx]

**Supplementary file 4**. Studies identified in literature

| **No.** | **Author (Year)** | **Title of Study** | **Inclusion/Exclusion** | **Reason for Exclusion** |
| --- | --- | --- | --- | --- |
| 1 | Abdullahi et al. (2016) | Traditional uvulectomy among neonates: experience in a Nigerian tertiary health institution. | Excluded | Objectives did not align with this review as its outcome variable differs. |
| 2 | Adebola et al. (2016) | Profile of pediatric traditional uvulectomy in North-West Nigeria: the need for caution and education. | Excluded | Low quality appraisal score and dropped as abstract reviewed |
| 3 | Adebola et al. (2016) | Profile of pediatric traditional uvulectomy in North-West Nigeria: the need for caution and education. | Excluded | Not meet the quality standard for inclusion criteria |
| 4 | (Adoga, 2011) | The traditionally amputated uvula amongst Nigerians: still an ongoing practice. | Included | Relevant to traditional uvulectomy. |
| 5 | Ajibade et al. (2013) | Harmful cultural practices: parents perceived effects of traditional uvulectomy on under-five children in Nigeria. | Included | Relevant to traditional uvulectomy. |
| 6 | Alebachew Bayih et al. (2020) | The burden of traditional neonatal uvulectomy among admissions to neonatal intensive care units, North Central Ethiopia. | Included | Relevant to traditional uvulectomy. |
| 7 | Baker et al. (2006) | Early initiation of and exclusive breastfeeding in large-scale community-based programmes in Bolivia and Madagascar. | Excluded | Focus on breastfeeding practices. |
| 8 | Che et al. (2017) | Traditional medicine. | Excluded | General focus on traditional medicine. |
| 9 | Faigel (1966) | Tonsillectomy—a bloody mess. | Excluded | Not related to traditional uvulectomy. |
| 10 | (Farouk et al., (2023) | Morbidity and mortality associated with traditional uvulectomy among neonates in a tertiary health facility in Kano. | Included | Relevant to traditional uvulectomy. |
| 11 | Gebrekirstos et al. (2013) | Magnitude and reasons for harmful traditional practices among children less than 5 years of age in Axum Town, Ethiopia. | Included | Relevant to traditional uvulectomy. |
| 12 | (Gebrekirstos et al., 2014) | A cross-sectional study on factors associated with harmful traditional practices among children less than 5 years. | Included | Relevant to harmful traditional practices. |
| 13 | Getachew et al. (2023) | The burdens, associated factors, and reasons of traditional uvulectomy in Ethiopia: A systematic review and meta-analysis. | Excluded | Not relevant to outcome variables, different objectives was there between studies |
| 14 | Haruna et al. (2023) | Determinants Of Reproductive Health Seeking Behaviour Among Women Of Reproductive Age In Ajaokuta Village, Kogi State. | Excluded | Not related to traditional uvulectomy. |
| 15 | Hunter (1995) | Uvulectomy—the making of a ritual. | Excluded | Not applicable |
| 16 | (Kambale *et al.*, 2018) | Traditional uvulectomy, a common practice in South Kivu in the Democratic Republic of Congo. | Included | Relevant to traditional uvulectomy. |
| 17 | Kebede et al. (2017) | Prevalence and associated factors to uvula cutting on under-five children in Amhara region, Debre Birhan town. | Included | Relevant to traditional uvulectomy. |
| 18 | Kefyalew et al. (2023) | Prevalence of traditional uvulectomy and its associated factors among parents with infants in Gondar City, Ethiopia. | Included | Relevant to traditional uvulectomy. |
| 19 | Kenu et al. (2021) | Factors that promote and sustain the use of traditional, complementary, and integrative medicine services. | Excluded | Focus on complementary medicine. |
| 20 | Kibira et al. (2023) | Uvula infections and traditional uvulectomy: Beliefs and practices in Luwero district, central Uganda. | Excluded | Qualitative study, Not relevant to outcome variables. |
| 21 | Latunji et al. (2018) | Factors influencing health-seeking behaviour among civil servants in Ibadan, Nigeria. | Excluded | Focus on health-seeking behavior. |
| 22 | Mitke (2010) | Bloody traditional procedures performed during infancy in the oropharyngeal area among HIV+ children. | Included | Relevant to harmful traditional practices. |
| 23 | Munn et al. (2023) | Assessing the risk of bias of quantitative analytical studies: introducing critical appraisal within JBI systematic reviews. | Excluded | Not related to traditional uvulectomy. |
| 24 | Musoke et al. (2014) | Health seeking behaviour and challenges in utilising health facilities in Wakiso district, Uganda. | Excluded | Focus on general health-seeking behavior. |
| 25 | Ndu et al. (2022) | Traditional Uvulectomy: A common and potentially life-threatening practice in a developing country. | Excluded | Not aligned with title of the study . |
| 26 | Olayinka et al. (2016) | Seroprevalence of hepatitis B infection in Nigeria: A national survey. | Excluded | Not related to traditional uvulectomy. |
| 27 | Owibingire et al. (2018) | Beliefs about traditional uvulectomy and teething: awareness and perception among adults in Tanzania. | Excluded | Not relevant to outcome variables and low quality under critical appraisals. |
| 28 | Prual et al. (1994) | Traditional uvulectomy in Niger: Epidemiology and consequences. | Excluded | Relevant to traditional uvulectomy. |
| 29 | Shaikh et al. (2005) | Health seeking behaviour and health service utilization in Pakistan: challenging the policy makers. | Excluded | General health-seeking behavior. |
| 30 | Yirdaw et al. (2022) | Practice and associated factors of traditional uvulectomy among caregivers having children less than 5 years old. | Included | Relevant to traditional uvulectomy. |
