# Supplementary material for: Pooled prevalence and associated factors of traditional uvulectom among children in Africa: A systematic review and meta-analysis
Source: PLoS One. 2025 Jan 28;20(1):e0316755. doi: 10.1371/journal.pone.0316755 (PMC11774377; doi:10.1371/journal.pone.0316755)
Supplement: Attachment — Submitted filename: Response to Reviewers comment 2(TCU)R2.docx [file pone.0316755.s007.docx]

Reviewer #1: A. Summary

The authors have stated in their reply that have addressed all the issues, however there are very few changes to the manuscript, many of the issues have not been addressed, and those that have been addressed have predominantly only been addressed in the response to reviewer and have not been added to the manuscript. In particular, the aims and objectives are still not clearly stated at the end of the background/introduction and the subheadings required by the PRISMA guidelines have still not been added to the abstract.

**Authors’ response**: it is added as ‘This study adhered to the ***PRISMA (Preferred Reporting Items for Systematic Reviews and Meta-Analyses)*** *guidelines to ensure transparency and reproducibility’* in the abstract and the objective also modified in the introduction section of the manuscript as ‘*This study aims to estimate the pooled prevalence of traditional childhood uvulectomy (TCU) practices in Africa’.*

The authors have not numbered the lines in their manuscript, which is a journal requirement and there has been little change to the multiple grammatical errors, which are too numerous to list as part of this review.
**Authors’ response:** revised by numbering lines and language editing.
I have listed the outstanding issues below, excluding all the grammatical errors, but considering the substantial and outstanding corrections required, I do not believe that this paper can be accepted.
**Authors’ response:** We revised thoroughly as much as possible and please reconsider your decision this paper to be accepted. We devoted our time and effort as much as all authors concerned.
B. Detailed issues
1. Abstract
1.1 The abstract is still missing multiple aspects which are required by PRISMA guidelines – the use of the subheadings listed in the PRISMA abstract check list is recommended, to ensure all aspects are included.

**Authors’ response**: revised
1.2 The following aspects must all still be addressed succinctly in the abstract.
1.2.1 The standard adhered to (PRISMA) should be stated.
1.2.2 The objectives need to be explicitly stated, describing the outcome (TCU)
1.2.3 The inclusion criteria need to be explicitly stated
1.2.4 The methods need to be briefly described.
1.2.5 The information sources/databases need to be stated
1.2.6 The risk of bias methods need to be stated
1.2.7 The characteristics of studies need to be stated
1.2.8 The limitations of the evidence included need to be stated
**Authors’ response:** revised by using it as per comments given
2. Introduction/Background
2.1 The statement: “Nevertheless, currently, in some parts of Sub-saharan Africa, as the majority of people depend on traditional medicine for their primary healthcare demands” appears to be missing some words and is not understandable as it stands.
**Authors’ response:** I did not get where that unclear statement is found. But, we tried to clarify each paragraph again in this revision.
2.2 The line, “A systematic intervention strategy in the current health care delivery system in Africa using the pooled prevalence and predictors of TCU could be launched”, needs to be rephrased as an aim and specific objectives must be stated, using the word, “objectives” : a clear statement at the end of the introduction stating aims and specific objectives is required.
**Authors’ response**: revised as ‘*The objective of this review and meta-analysis was estimating pooled prevalence and predictors of traditional childhood uvulectomy (TCU) practices in Africa.’*
3. Methods
3.1 Add or clarify the following aspects:
3.1.1 The statement, “Subgroup analysis was performed by considering Ethiopia and other African countries, study settings, and sample size categories to overcome the inflation of the pooled effect from the inclusion of studies.” Should more precisely and clearly state the exact subgroups.

**Authors’ response:** *Subgroup analysis was conducted based on the following factors: country (Ethiopia, which had a higher concentration of primary studies, and other African countries), study setting (community-based or health facility-based), and sample size (categorized as less than 500 or 500 and above).*
3.1.2 The start date stated in the author response must be included in the methods section of the manuscript.

**Authors’ response:** add on the manuscript (the start date).
3.2 Search strategy and quality assessment
3.2.1 The statement “The meta-analysis also included studies published in English and studies conducted only in African countries.” Needs to be rephrased to more clearly state that ONLY studies published in English and in Africa were included.
**Authors’ response**: corrected
3.2.2 The last line of the paragraph with title, “Study selection: states, “Two independent authors subsequently reviewed the abstracts and full texts of eleven articles” – the initials of those authors must be stated.

**Authors’ response:** revised as ‘*Two independent authors (TMA and AK) reviewed the abstracts and full texts of eleven articles published between 2010 and 2024.’*
3.2.3 The search strategy was only provided for Medline and is not complete, since no terms for uvulectomy were described. The full search strategy for all databases is required by PRISMA standards.

**Authors’ response:** we used similar the search strategies to all database by using different Boolean operators mentioned in the manuscript.
3.2.4 There is insufficient detail in the main text of the manuscript summarising how risk of bias was assessed – the reader should not have to refer to supplementary files.
**Authors’ response:** revised
3.3 Outcome measurement and statistical analysis
3.3.1 Under outcome measurement, the statement, “all the procedures of cutting off a part of the uvula by traditional healers in the childhood age group (birth to under 15) in all African countries” appears to be missing a section – this has not been corrected as requested - please edit and clarify.
**Authors’ response:** revised as ‘’ *Traditional Childhood Uvulectomy (TCU) is a cultural practice in which a portion of the uvula (the small, fleshy extension at the back of the soft palate) is surgically removed or altered by traditional healers or unqualified practitioners, typically without medical supervision on under 15 age group in Africa*.’’
3.3.2 Please provide a summary of the rationale for using the weighted inverse variance random-effects model in the text of the manuscript.
**Authors’ response**: add a reason as ‘*This model was selected because it is better suited to handle the variability between studies, providing a more nuanced and reliable estimate of the pooled prevalence of TCU and its predictors across Africa, compared to a fixed-effects model’.*

3.3.3 The statement, “considering Ethiopia and other African countries” should be edited in the text to, “comparing Ethiopia to other African countries”
**Authors’ response**: revised
3.3.4 Please state, in the manuscript, the rationale for grouping Ethiopia separately to other African countries.
**Authors’ response:** as there is a number of studies in Ethiopia which is comparable to other countries combined.
3.3.5 Please provide a brief clear statement in the manuscript in the methods, stating which categories were used for synthesis and why?
**Authors’ response**: *Subgroup analysis was conducted based on the following factors: country (Ethiopia, which had a higher concentration of primary studies, and other African countries), study setting (community-based or health facility-based), and sample size (categorized as less than 500 or 500 and above).* As this is the possible category to have a maximum possibility to balance the study participants in primary studies, we used the aforementioned category.
3.3.6 Please briefly describe in the manuscript, how were missing summary statistics were handled.

3.3.7 Please succinctly describe in the manuscript, what methods were used to tabulate or visually display results of individual studies and syntheses.
**Authors’ response:** tables and figures
3.3.8 Please succinctly describe in the manuscript what methods were used to assess risk of bias due to missing results in a synthesis (not the same as publication bias)
**Authors’ response**: we tried to show it by publication bias-Eger’s test. And we took care of searching of literatures robustly and in addition doing sensitivity analysis to show each studies effect on the overall pooled prevalence.

3.3.9 Please describe in the manuscript, what p-values were used to interpret the publication bias assessment.
**Authors’ response:** Publication bias assessed by Eger’s’ test. The absence of publication bias was assessed using Egger’s regression test. A significant result (P<0.05) suggests potential publication bias, while a non-significant result (P>0.05) indicates no evidence of bias. In this analysis, Egger’s test yielded p = 0.098, indicating no significant evidence of publication bias.

4. Results
4.1 Figure 1
The revised figure 1 was not included – please supply it to determine if the below amendments were done.
4.1.1 The text in box, “The title and abstract do not fit” should be reworded more scientifically (eg “Title and/or abstract not relevant”)
4.1.2 The numbers of records per data base should be stated in the figure
**Authors’ response:** is that mean the number of articles found, is so, it is considered
4.2 Characteristics of included studies
4.2.1 Only seven studies in Ethiopia were identified, compared to 19 in the review by Getachew et al. (International Journal of Pediatric Otorhinolaryngology 176 (2024) 111835), despite the same eligibility criteria – please explain the discrepancy to the reviewer and ensure that this is discussed in the discussion. The authors need to address the above question factually. In their response to the reviewer, they speculate what the possible reasons may be, however that is not acceptable – they need to thoroughly read the paper and clearly and factually explain in the manuscript, the reasons for the differences, in the discussion section of their manuscript.
**Authors’ response:** We reviewed the paper but did not find a definitive answer to the question. As previously mentioned there is considerable variability in **both objectives and timing across studies=the objective for Getachew et al 2024 study is for complication burden secondary to uvulectomy not the pooled prevalence. (**[**https://www.cell.com/heliyon/fulltext/S2405-8440(24)15009-8?uuid=uuid%3Ac15fd92c-c193-4caa-b8c3-ed7d45f37f0c)**](https://www.cell.com/heliyon/fulltext/S2405-8440(24)15009-8?uuid=uuid%3Ac15fd92c-c193-4caa-b8c3-ed7d45f37f0c))**.** what is more confused about it is in other journal it seems pooled prevalence (file:///C:/Users/HAB-TECH/Downloads/Uvlectomy%202.pdf). While we excluded qualitative studies, the study **by Getachew et al. incorporated mixed-method/title studies**. *For example, it included research assessing knowledge of neonatal care, where uvulectomy was mentioned as one aspect but was not the primary focus. Getachew et al. also included articles exploring knowledge and attitudes, which often relied on qualitative methods to investigate reasons for uvulectomy.*

**In contrast, our review primarily focused on the practice of uvulectomy and its associated factors. As a result, we excluded several studies included by Getachew et al**. For instance, Hadush A., 2016 (titled “Assessment of Knowledge and Practice of Neonatal Care among Postnatal Mothers Attending Ayder and Mekelle Hospital in Mekelle, Tigray, Ethiopia 2013”) was excluded from our review because it primarily addressed neonatal care rather than uvulectomy.

Additional factors explain the differences between our review and that of Getachew et al. First, we included only studies scoring 6 or above out of 9 on our quality appraisal checklist. Second, we focused on articles that reported at least one associated factor or the prevalence of uvulectomy. Third, Getachew et al. may have included preprints or unpublished studies, whereas we restricted our review to published articles to ensure quality assurance.

Finally, differences in population scope may also contribute to discrepancies. Our review specifically targeted childhood uvulectomy (ages birth to 15 years), whereas Getachew et al.’s inclusion criteria may have encompassed broader populations or varying age groups.

4.2.2 The table listing the characteristics of included studies should be referenced at the beginning of the paragraph, not at the end.
**Authors’ response:** corrected
4.2.3 In the manuscript, the term, “risk of bias” should be used in preference to “risk”.
**Authors’ response**: revised
4.2.4 The term “included” should be used rather than “considered”, since all were included.
**Authors’ response:** considered
4.2.5 Please add the word, “all” to the statement (as underlined ) , “none of the included studies were of poor quality and all had a quality score ≥6 out of 9 criteria”
**Authors’ response:** revised
4.2.6 Please edit “JBI quality appraisal checklist,” to “JBI risk of bias quality appraisal checklist”
4.2.7 The 9 quality criteria should be stated, and the scores should be included separately in a graphic. Supplementary file 2 should be included in the mian text of the manuscript as a table, with “yes” “no” “low risk” presented as graphics, rather than text.
**Authors’ response:** as it is checklist, it makes the reader unpleasant I think. If it is must, I will do.

Table 2: Quality Assessment of the Included Studies Using JBI Risk of Bias Assessment checklist

|  | **Criteria** | | | | | | | | | Score | Quality |
| --- | --- | --- | --- | --- | --- | --- | --- | --- | --- | --- | --- |
| **Author** | Was The Sample Frame Appropriate? | Was Sampling Appropriate? | Was The Sample Size Adequate? | Were The Study Subjects And The Setting Described In Detail? | Was The Data Analysis Conducted With Sufficient Coverage Of The Identified Sample? | Were Valid Methods Used For The Identification Of The Condition? | Was The Condition Measured In A Standard, Reliable Way For All Participants? | Was There Appropriate Statistical Analysis? | Was The Response Rate Adequate, And If Not, Was The Low Response Rate Managed Appropriately? |  |  |
| (Farouk et al., (2023) | Yes | Yes | Yes | Yes | Yes | Yes | Yes | Yes | Yes | 9 | Low risk |
| (Kambale *et al.*, 2018) | yes | yes | no | yes | no | yes | yes | yes | no | 6 | Low risk |
| (Adoga, 2011) | yes | yes | yes | no | yes | yes | no | yes | yes | 7 | Low risk |
| (Oluwatosin *et al.*, 2016) | yes | yes | yes | yes | yes | yes | yes | yes | yes | 9 | Low risk |
| Djakounda, 1994) | yes | yes | yes | yes | not clear | yes | yes | yes | yes | 8 | Low risk |
| (Gebrekirstos et al., 2014) | yes | yes | yes | not clear | yes | yes | yes | yes | yes | 8 | Low risk |
| (Mitke, 2010) | yes | yes | yes | yes | yes | yes | yes | yes | yes | 9 | Low risk |
| (Kebede *et al.*, 2017) | yes | yes | yes | yes | yes | yes | no | yes | no | 6 | Low risk |
| (Yirdaw et al., 2022) | yes | yes | no | yes | yes | no | no | yes | yes | 6 | Low risk |
| (Gebrekirstos et al., 2014) | yes | yes | yes | yes | no | yes | yes | no | no | 6 | Low risk |
| (Bayih et al., 2020) | yes | yes | yes | yes | yes | yes | yes | yes | yes | 9 |  |

4.2.8 Please add a table in the manuscript with details of the ten excluded studies which “did not report the outcome of interest”.
**Authors’ response:** added excluded studies of this manuscript as Table 2.
4.3 Meta-analysis
4.3.1 It is more intuitive to the reader to first read about the pooled incidence of TCU, subgroup analysis and predictors , before describing the Egger’s test, so it is clear what outcomes it refers to.
4.3.2 Egger’s test assesses publication bias, not “absence of publication bias” – the text should briefly explain how the parameters in table show no publication bias.
**Authors’ response:** revised
4.3.3 The title of table 2 could rather be stated as, “Egger’s test for publication bias in the effect sizes of prevalence and predictors of TCU in Africa.”
**Authors’ response:** revised as per comments lines 17-22.
4.3.4 What is meant by, “mothers with a lack of information and who were perceived as
uvula causes”? Please clarify in the manuscript.
**Authors’ response:** revised as’’ *In a single study by Yirdaw et al., mothers who lacked information about uvulectomy and those who perceived the uvula as a cause of infection were significantly more likely to practice traditional childhood uvulectomy (TCU) compared to those who were informed about its complications’’.*
4.4 Subgroup analysis

4.4.1 Please add more more detail in the manuscript describing exactly which African countries, types of communities and types of health facilities were grouped.
**Authors’ response:** revised in lines from 25-36 on page 13.
4.4.2 The interpretation of the results with statements such as, “This implies that nearly half of the children would not seek medical care after they had received TCU”, should be moved from the results section to the discussion.
**Authors’ response**: revised
4.4.3 Table 3 needs a more detailed title explaining what it represents. Needs footnotes with abbreviations and explanations of the subgroups – eg, which other African countries, what does community-based mean? What does health facilities mean?
**Authors’ response:** revised
4.4.4 The associated forest plots for the subgroup analyses should be included in the text (these are different to figures 2 – 4, and could not be found by the reviewer)

4.5 Predictors of TCU
4.5.1 Please correct the grammar of the statement, “nearly fourfold more patients with uvulitis not cured by medical treatment not cured by medical treatment than did their counterparts”
4.5.2 Please correct the grammar/syntax of the statement, in the last line of the results, “AOR=4.31; 95% CI=2.17-6[8,9]/>, respectively).”
**Authors’ response**: Revised
5. Discussion
5. 1 The discussion should begin with statement referring to what was described in the study, rather than repeating the aim.
5.2 Please discuss the results for the Ethiopian studies in detailed comparison to the systematic review of TCU in Ethiopia by Getachew et al.- see 4.2.1

**Authors’ response**: we used it however, the study by Getachew is not clear to use as it reported as *prevalence* in **International Journal of Pediatric Otorhinolaryngology** **Journal** and ***The pooled incidence of complications following traditional uvulectomy* in Heliyon Journal.**

5.3 The predictors of TCU were rural residency and a history of previous practice – please discuss in the text of the manuscript, why maternal education was not included, since this was identified in the more comprehensive systematic review of TCU in Ethiopia by Getachew et al.?
**Authors’ response**: We did not get an association education with uvulectomy.
5.4 The statement “the most common utilizers of traditional medicine were found in India, China, and Africa”, appears to refer to the current study, which was limited to Africa – please reword the statement in your manuscript to clarify which studies it refers to.
**Authors’ response:** revised
5.5 Please add a paragraph at the end of the discussion in the manuscript describing the strengths and limitations of your study.
**Authors’ response**: added
5.6 Please add a paragraph in the conclusion of your manuscript, clearly, precisely and succinctly explaining how the findings of your study can be used to develop a project addressing TCU?

**Authors’ response**: revised

Reviewer #2: (No Response)

Reviewer #3: Thank you for the opportunity to rearview the text.
The authors attempted to address some comments provided by the reviewers.
I remain of the opinion that the text needs to be screened in depth for grammar, punctuation, tenses and complete sentences and use of correct phrases. Reviewer #3 made several comments/suggestions to improve this, which are not adequately addressed, but which might have improved clarity for the reader.
The text still implies, in part, that mothers/parents either practice TCU themselves or receive TCU themselves, instead of their children.
Search criteria and results criteria for platforms searched are incongruent. Also, no results were highlighted for Science Direct. If necessary, a PRISMA search diagram may be included in the text (as opposed to a supplement) to improve the section for search/results of literature.
Reviewer #2 requested search results be included into the Abstract.
Please check consistency of spelling for "sub-Saharan"

**Authors’ response**: revised as much as we can.

Thank you again.
