# Supplementary material for: Pooled prevalence and associated factors of traditional uvulectom among children in Africa: A systematic review and meta-analysis
Source: PLoS One. 2025 Jan 28;20(1):e0316755. doi: 10.1371/journal.pone.0316755 (PMC11774377; doi:10.1371/journal.pone.0316755)
Supplement: Attachment — Submitted filename: PONE-D-24-36948_reviewer.docx [file pone.0316755.s005.docx]

**PLOS ONE**

**Prevalence of traditional uvulectomy and associated predictors among children in Africa: A systematic review and meta-analysis**

--Manuscript Draft--

| **Manuscript Number:** | PONE-D-24-36948 |
| --- | --- |
| **Article Type:** | Research Article |
| **Full Title:** | Prevalence of traditional uvulectomy and associated predictors among children in Africa: A systematic review and meta-analysis |
| **Short Title:** | Traditional uvulectomy among children in Africa: A systematic review and meta- analysis |
| **Corresponding Author:** | Solomon Demis  Debre Tabor University  Debre Tabor, South Gondar ETHIOPIA |
| **Keywords:** | malpractice; uvulectomy; traditional practices; traditional healer; traditional medicine |
| **Abstract:** | Background  Traditional childhood uuvlectomy(TCU) is an unsupported, unsupervised, and possibly a dangerous cultural malpractice. It is often accompanied by life-threatening conditions such as infection, anaemia, aspiration, and oral-pharyngeal injuries caused by the use of unsafe instruments of blades, needles, or thread loops. These instruments are usually used on many children in the same session, thereby increasing the risk of transmitting communicable diseases, like HIV and hepatitis B virus.  Design  A weighted inverse-variance random effects model was used to compute the overall pooled prevalence and predictors. Variations across the studies were checked using a forest plot, I2 statistics, and Egger's test.  Setting  Countries in Africa.  Participants  Eleven primary studies with a sample size of 7231 children. Outcome measures  TCU is all the procedures for cutting a part of the uvula by traditional healers in the childhood age group (birth to under15) in all African countries.  Results  A total of 11 studies with a sample of 7231 children were included in this meta- analysis. The pooled estimate of TCU in Africa was 40.98% (95% CI=25.04-56.92; I2=99.61, P=0.001). Mothers who lived in rural areas were 2.45 times more likely to have a child with TCU than those who lived in urban areas (AOR=2.45; 95% CI=1.59- 3.32). Mothers with a history of previous TCU practice were 8.44 times more likely to practice than those with no history (AOR=8.44; 95% CI=6.27-10.61).  Conclusion  More than two-fifths out of 7231 children in Africa were uvulectomized traditionally. Fortunately, all uvulectomy practices did not cause hospitalization, as shown by the discrepancies between studies by community and health facilities. The predictors of TCU were rural residency and a history of previous practice. Therefore, the problem should be prioritized by the current maternal child health packages in African countries. |

|  | Prospero ID CRD42024498699. |
| --- | --- |
| **Order of Authors:** | Solomon Demis Kebede |
|  | Kindu Agmas |
|  | Demewoz Kefale |
|  | Amare Kassaw |
|  | Tigabu Munye Aytenew |
| **Additional Information:** |  |
| **Question** | **Response** |
| **Financial Disclosure**  Enter a financial disclosure statement that describes the sources of funding for the work included in this submission. Review the [submission guidelines](http://journals.plos.org/plosone/s/submission-guidelines#loc-financial-disclosure-statement) for detailed requirements. View published research articles from [*PLOS ONE*](https://journals.plos.org/plosone/) for specific examples.  This statement is required for submission and **will appear in the published article** if the submission is accepted. Please make sure it is accurate. | The author(s) received no specific funding for this work. |
| **Funded studies**  Enter a statement with the following details:   - Initials of the authors who received each award - Grant numbers awarded to each author - The full name of each funder - URL of each funder website - Did the sponsors or funders play any role in the study design, data collection and analysis, decision to publish, or preparation of the manuscript? |  |
| Did you receive funding for this work? |  |
| **Competing Interests**  Use the instructions below to enter a competing interest statement for this submission. On behalf of all authors, disclose any [competing interests](http://journals.plos.org/plosone/s/competing-interests) that could be perceived to bias this work—acknowledging all financial support | The authors have declared that no competing interests exist |

| and any other relevant financial or non- financial competing interests.  This statement is required for submission and **will appear in the published article** if the submission is accepted. Please make sure it is accurate and that any funding sources listed in your Funding Information later in the submission form are also declared in your Financial Disclosure statement.  View published research articles from  [*PLOS ONE*](http://journals.plos.org/plosone/) for specific examples. |  |
| --- | --- |
| **NO authors have competing interests**  Enter: *The authors have declared that no competing interests exist.*  **Authors with competing interests**  Enter competing interest details beginning with this statement:  *I have read the journal's policy and the authors of this manuscript have the following competing interests: [insert competing interests here]* |  |
| * typeset |  |
| **Ethics Statement**  Enter an ethics statement for this submission. This statement is required if the study involved:   - Human participants - Human specimens or tissue - Vertebrate animals or cephalopods - Vertebrate embryos or tissues - Field research   Write "N/A" if the submission does not require an ethics statement.  General guidance is provided below. | Not applicable as it is systematic review and meta-analysis |

| Consult the [submission guidelines](http://journals.plos.org/plosone/s/submission-guidelines#loc-guidelines-for-specific-study-types) for detailed instructions. **Make sure that all information entered here is included in the Methods section of the manuscript.** |  |
| --- | --- |
| **Format for specific study types**  **Human Subject Research (involving human participants and/or tissue)**   - Give the name of the institutional review board or ethics committee that approved the study - Include the approval number and/or a statement indicating approval of this research - Indicate the form of consent obtained (written/oral) or the reason that consent was not obtained (e.g. the data were analyzed anonymously)   **Animal Research (involving vertebrate animals, embryos or tissues)**   - Provide the name of the Institutional Animal Care and Use Committee (IACUC) or other relevant ethics board that reviewed the study protocol, and indicate whether they approved this research or granted a formal waiver of ethical approval - Include an approval number if one was obtained - If the study involved *non-human primates*, add *additional details* about animal welfare and steps taken to ameliorate suffering - If anesthesia, euthanasia, or any kind of animal sacrifice is part of the study, include briefly which substances and/or methods were applied   **Field Research**  Include the following details if this study involves the collection of plant, animal, or other materials from a natural setting:   - Field permit number - Name of the institution or relevant body that granted permission |  |
|  |  |
| **Data Availability**  Authors are required to make all data underlying the findings described fully available, without restriction, and from the | Yes - all data are fully available without restriction |

| time of publication. PLOS allows rare exceptions to address legal and ethical concerns. See the [PLOS Data Policy](http://journals.plos.org/plosone/s/data-availability) and [FAQ](http://journals.plos.org/plosone/s/data-availability#loc-faqs-for-data-policy) for detailed information.  A Data Availability Statement describing where the data can be found is required at submission. Your answers to this question constitute the Data Availability Statement and **will be published in the article**, if accepted. |  |
| --- | --- |
| **Important:** Stating ‘data available on request from the author’ is not sufficient. If your data are only available upon request, select ‘No’ for the first question and explain your exceptional situation in the text box. |  |
| Do the authors confirm that all data underlying the findings described in their manuscript are fully available without restriction? |  |
| **Describe where the data may be found in full sentences. If you are copying our sample text, replace any instances of XXX with the appropriate details.**   - If the data are **held or will be held in a public repository**, include URLs, accession numbers or DOIs. If this information will only be available after acceptance, indicate this by ticking the box below. For example: *All XXX files are available from the XXX database (accession number(s) XXX, XXX.*). - If the data are all contained **within the manuscript and/or Supporting Information files**, enter the following: *All relevant data are within the manuscript and its Supporting Information files.* - If neither of these applies but you are able to provide **details of access elsewhere**, with or without limitations, please do so. For example:   *Data cannot be shared publicly because of [XXX]. Data are available from the XXX Institutional Data Access / Ethics Committee (contact via XXX) for* | The data will be available upon request to corresponding author (solomondemis@gmail.com). |

| *researchers who meet the criteria for access to confidential data.*  *The data underlying the results presented in the study are available from (include the name of the third party and contact information or URL).*   - This text is appropriate if the data are owned by a third party and authors do not have permission to share the data.   * typeset |  |
| --- | --- |
| Additional data availability information: |  |

Tradtional children uvulectomy in Africa	[Click here to access/download;Manuscript;TCU SR and MA1.DOCX](https://www2.cloud.editorialmanager.com/pone/download.aspx?id=37359255&guid=468027c1-2f1b-406e-bc4d-af19d232fa00&scheme=1)

# Prevalence of traditional uvulectomy and associated predictors among children in Africa: A systematic review and meta-analysis

**Solomon Demis Kebede^1*^, Kindu Agmas^2^, Demewoz Kefale^3^, Amare Kassaw^3^, Tigabu Munye Aytenew^4^**

**1** Department of Maternity and Neonatal Nursing, College of Health Sciences, Debre Tabor University, Debre Tabor, Ethiopia

**2** Department Pediatrics and Child Health, Debre Tabor Comprehensive Specialized Hospital, Debre Tabor, Ethiopia

**3** Department of Pediatrics and Child Health Nursing, College of Health Sciences, Debre Tabor University, Debre Tabor, Ethiopia

**4** Department of Nursing, College of Health Sciences, Debre Tabor University, Debre Tabor, Ethiopia

***Corresponding author**:

Solomon Demis Kebede

Department of Maternity and Neonatal Nursing, College of Health Sciences, Debre Tabor University, Debre Tabor, Ethiopia

[solomondemis@gmail.com](mailto:solomondemis@gmail.com)

# Abstract

**Background:** Traditional childhood uvulectomy is an unsupported, unsupervised, and possibly a dangerous cultural malpractice. It is often accompanied by life-threatening conditions such as infection, anaemia, aspiration, and oral-pharyngeal injuries caused by the use of unsafe instruments of blades, needles, or thread loops. These instruments are usually used on many children in the same session, thereby increasing the risk of transmitting communicable diseases, like HIV and hepatitis B virus.

**Design:** A weighted inverse-variance random effects model was used to compute the overall pooled prevalence and predictors. Variations across the studies were checked using a forest plot, I^2^ statistics, and Egger's test.

**Setting**: Countries in Africa.

**Participants:** Eleven primary studies with a sample size of 7231 children.

**Outcome measures**: Traditional Childhood uvulectomy is all the procedures for cutting a part of the uvula by traditional healers in the childhood age group (birth to under15) in all African countries.

**Results**: A total of 11 studies with a sample of 7231 children were included in this meta- analysis. The pooled estimate of TCU in Africa was 40.98% (95% CI=25.04-56.92; I^2^=99.61, P=0.001). Mothers who lived in rural areas (AOR=2.45; 95% CI=1.59-3.32), Mothers with a history of previous TCU practice (AOR=8.44; 95% CI=6.27-10.61) were significantly associated with (Add your Outcome).

**Conclusion:** More than two-fifths out of 7231 children in Africa were uvulectomized traditionally. Fortunately, all uvulectomy practices did not cause hospitalization, as shown by the discrepancies between studies by community and health facilities. The predictors of TCU were rural residency and a history of previous practice. Therefore, the problem should be prioritized by the current maternal child health packages in African countries.

**Prospero ID**: CRD42024498699.

**Keywords**: malpractice, uvulectomy, traditional practices, Children, traditional healer, traditional medicine, Africa

# Background

As part of traditional medicine in Africa, traditional uvulectomy is performed on the anatomical structure of the upper respiratory tract called the Uvula. It is a small soft tissue that hangs down from the back of the mouth above the throat between the two tonsils. It has the natural advantages of preventing aspiration, lubricating oropharyngeal mucosa, serving language communication, boosting immunological function, and preventing breast milk regurgitation through the nose [1–4].

Traditional medicine is defined by the World Health Organization (WHO) as knowledge, skills, and practices based on theory, beliefs, and experiences framed by indigenous culture in the maintenance of health, in the prevention of disease, or for treatment purposes. Some of these procedures are supported by modern scientific evidence, and some are harmful to the health of the community even if they are practiced, such as childhood traditional uvulectomy. Currently, in some parts of the world, including Africa, the majority of people depend on traditional medicine for their primary healthcare demands[5,6].

Traditional childhood uvulectomy is an unsupported, illogical, and possibly a dangerous cultural malpractice procedure for all childhood sickness, including feeding, breathing, and related diseases. It is often accompanied by life-threatening neonatal morbidities such as infection, septicaemia, anaemia, aspiration, and oral-pharyngeal injuries caused by the use of unsafe instruments such as blades, needles, or thread loops. These instruments are usually used on many children in the same session, thereby increasing the risk of transmitting communicable diseases, mainly HIV and hepatitis virus[4,7,8].

Moreover, other common complications of traditional children uvulectomy consists of anaemia, haemorrhage, sepsis, tetanus, aspiration and pain for many days after the procedure. Changes in voice, disturbances in sleep patterns and regurgitation of breast milk from the nostril are also complications after uvulectomy practices. Thus, childhood admissions attributed to these complications require antibiotics, oxygen, intravenous fluid, blood transfusion, phototherapy, and a greater number of health care providers, thereby accelerating the cost in the health care system [3,9,10].

Childhood uvulectomy is traditionally an old cultural component of surgery in many countries in Africa. The procedure persists in developing countries, probably because of low socioeconomic status and non-formal education level. In the African context, the community mistakenly attributed nearly all childhood illnesses to uvula so that ill children are often subjected to traditional uvulectomy because of parental perception of poor outcomes in modern medicine.

Morbidities resulting from post-uvulectomy complications contributed to the high burden of under-five childhood mortality in Africa. This is because, unlike other harmful traditional practices, traditional childhood uvulectomy is still being practiced in Africa.

As per studies on childhood traditional uvulectomy in several African countries individually; it affects several children through the cutting of a part of the uvula by traditional healers. Despite its significant clinical practical benefits, the pooled prevalence of TCU practice and predictors for TCU in Africa were not appropriately incorporated in the prevention of neonatal and under five mortality. Furthermore, there are no current systematic reviews or meta-analyses on this topic. A systematic intervention strategy in the current health care delivery system in Africa using the pooled prevalence and predictors of TCU could be launched.

# Methods and Materials

## Reporting

The results of this review were reported based on the Preferred Reporting Items for Systematic Review and Meta-analysis (PRISMA) statement guidelines (**supplementary file 1**). PROSPERO registration can be accessed at (**http[s://w](http://www.crd.york.ac.uk/prospero/#myprospero))ww.c[rd.york.ac.uk/prospero/#myprospero](http://www.crd.york.ac.uk/prospero/#myprospero))**[).](http://www.crd.york.ac.uk/prospero/#myprospero))

## Search strategy and information source

The adapted PICO format was used to explicitly review the literature and clarify the specifications of the inclusion and exclusion criteria. The adapted PICO comprises population (P), exposure (E), outcome (O), and context (setting as described below).

a. **Population**: children
b. **Exposure**: Associated factors, predictors, risk factors
c. **Outcome**: traditional uvulectomy
d. **Context (Setting):** African countries.

Therefore, by using this adapted PICO format, the following review questions were formulated for the search for primary studies.

1. What is the pooled incidence of traditional childhood uvulectomy in Africa?
2. What are the predictors associated with traditional childhood uvulectomy in Africa? Based on the aforementioned review PICO format and questions, primary studies performed from MEDLINE, Science Direct, Google Scholar, and African Journal Online were searched.

The core search terms and phrases used were “prevalence”, "incidence", “epidemiology”, “proportion”, “magnitude”, “burden”, “predictors”, “risk factors”, “associated factors” and “Africa”. The search strategies were developed using different Boolean operators. Notably, to fit the advanced MEDLINE database, the following search strategy was applied until December 23, 2023: [(prevalence[MeSH Terms]) OR incidence[MeSH Terms]) OR proportion [MeSH Terms]) OR epidemiology[MeSH Terms]) OR magnitude[MeSH Terms]) OR burden[MeSH Terms]) AND predictors [MeSH Terms]) OR risk factors [MeSH Terms]) OR associated factors [MeSH Terms]) AND (Africa)].

## Study selection

Primary studies downloaded and retrieved from the databases were exported to Mendeley Desktop 1.19.8 reference manager software to remove duplicate studies. The study selection process was held in two stages. First, the title and abstract were screened, and second, a full-text review was performed. Two independent reviewers (KA and DK) screened the title and abstract. Disagreements were resolved based on established article selection criteria, and discussion with the third author (SD) resolved any disagreements to decide whether the study was eligible for inclusion or not. Two independent authors subsequently reviewed the abstracts and full texts of eleven articles.

## Eligibility criteria

**Inclusion criteria**: Included studies were articles that reported the prevalence of traditional childhood uvulectomy in general and/or at least one or more predictors in Africa. The meta-

analysis also included studies published in English and studies conducted only in African countries.

**Exclusion criteria:** Articles without full text available, adult uvulectomy, or qualitative studies were excluded.

**Quality assessment**

**Critical appraisal and quality assessment**

We used the quality appraisal criteria of the Joanna Briggs Institute (JBI)[11]. Studies were considered low risk when they fit quality assessment checklists of 6 or above. Two independent authors (SD and DK) appraised the quality of the studies. Disagreements were resolved by consulting a third reviewer (KA). The quality scores for the primary studies were reported (**Supplementary file 2**).

## Data extraction

The data were determined to be extracted based on two criteria: 1. Clear and consistent operational definitions for the dependent variable (traditional childhood uvulectomy) and 2. These variables are statistically associated with the outcome variable reported by the AOR. Two authors extracted the data using the standardized format of the MS Excel spread sheet. The name of the first author and year, country name, study setting, study design, sample size, sample category, prevalence, and odds of predictors were extracted. Whenever variations are observed, the phase is repeated. If discrepancies between the data extracted were present, a third reviewer was involved in the decision (SD). After reporting the inconsistency of the data in the primary study, data transformation was conducted.

# Outcome measurement

## Traditional childhood uvulectomy

All the procedures of cutting off a part of the uvula by traditional healers in the childhood age group (birth to under 15) in all African countries [3,12,13].

## Statistical analysis

The required data were collected using the Microsoft Excel 2013 workbook. Then, STATA version 17 statistical software was used for the meta-analysis. Publication bias was objectively checked using Egger’s regression test. The heterogeneity of the studies was quantified using the I-squared statistic. Both pooled prevalence analysis and pooled effect of predictors were conducted using a weighted inverse variance random-effects model. Subgroup analysis was performed by considering Ethiopia and other African countries, study settings, and sample size categories to overcome the inflation of the pooled effect from the inclusion of studies.

# Results

## Selection of studies for review

The search strategy retrieved 46 articles, 16 of which were identified as duplicates and removed. Among the screened articles, 30 were from PubMed (n=12), Google Scholar (n=10), and the African Health Journal Repository (n=8). The full texts of twenty-one articles were reviewed, and eleven studies were included in this review and meta-analysis (Figure 1).

## Characteristics of the included studies

A total of eleven cross-sectional studies with a pooled sample size of 7,231 children were included in this meta-analysis. Seven studies were found in each part of Ethiopia, particularly two from the Northwest [1,3], two from the North [14,15], one from the Central Hemisphere [16], two from the Southern Hemisphere [17,18], two from Nigeria in the Northwest and Plateau states [19,20] and one each from the Democratic Republic of the Congo and Niger[8,21].

The maximum sample size was 1163, and the minimum sample size was 402 among the reviewed studies, for a total of 7231 children [17,18]. Traditional childhood uvulectomy was not practiced across Africa. This practice is more common in some places than in others according to this review. Regarding this unequal distribution, the study settings for articles from Ethiopia were as follows: two articles from the Northwest, particularly from Debre Tabor[1,3]; two from southern Hawassa and Gamo zone[17,18]; two from northern Axum town by[15]; and one from central region in Debre Birhan town [16].

One study from another part of Africa was from the DRC in South Kivu[8], two studies from Nigeria in the Northwest region and plateau state[19,20], and one study from Niger in Niamey[21].

The studies included in this systematic review and meta-analysis had no considerable risk. Therefore, all the studies were considered. We assessed the studies with a JBI quality appraisal checklist for systematic review[11]. According to this appraisal checklist, none of the included studies were of poor quality and had a quality score ≥6 out of 9 criteria (**Table 1**).

**Table 1**. Characteristics of the studies reviewed on the prevalence and predictors of TCU in Africa.

| **No** | **Author** | **Country** | **Study setting** | **Sample size**  **category** | **Study design** | **Age group** | **Region category** | **Study area** |
| --- | --- | --- | --- | --- | --- | --- | --- | --- |
| 1 | Adoga, (2011) | Nigeria | health facility  based | >500 | cross-sectional | under 10 | Nigeria | Jos |
| 2 | Bayih et al., (2020) | Ethiopia | health facility  based | <500 | cross-sectional | neonates | Northwest | Debre  Tabor |
| 3 | Djakounda, (1994) | Niger | health facility  based | >500 | cross-sectional | under 5 | Central | Niamey |
| 4 | Farouk et al., (2023) | DRC | health facility  based | >500 | cross-sectional | under 15 | South Kivu | South Kivu |
| 5 | Gebrekirstos et al.,  (2013) | Ethiopia | community-based | >500 | cross-sectional | under 5 | Northern | Axum |
| 6 | Gebrekrstos et al.,  (2014) | Ethiopia | community-based | >500 | cross-sectional | under 5 | Northern | Axum |
| 7 | Kebede et al., (2017) | Ethiopia | community-based | <500 | cross-sectional | under 5 | Central | Debre  Birhan |
| 8 | Kefelew et al., (2023) | Ethiopia | health facility  based | <500 | cross-sectional | under 5 | Southern | Gamo |
| 9 | Mike, (2010) | Ethiopia | community-based | >500 | cross-sectional | under 5 | Southern | Hawassa |
| 10 | Oluwatosin et al.,  (2016) | Nigeria | health facility  based | >=500 | cross-sectional | under 15 | Northwest | Nigeria |
| 11 | Yirdaw et al (2022) | Ethiopia | community-based | >500 | cross-sectional | under 5 | Northwest | South  Gondar |

# Meta-analysis

## The pooled incidence of TCU

The absence of publication bias was assessed with Egger’s regression test analysis (p = 0.098), which showed no publication bias (**Table 2**).

**Table 2**: Egger’s test for the prevalence and predictors of TCU in Africa

**Std.-Eff. Coef.	Std. Err	t	p>t	95% confidence interval**

| **Slope** | -12.06589 | 18.557902 | -0.65 | 0.532 | -54.04932 | 29.91754 |
| --- | --- | --- | --- | --- | --- | --- |
| **Bias** | 62.57097 | 33.95122 | 1.84 | **0.098** | -.14.23202 | 139.374 |

**Pooled incidence of TCU**

The pooled incidence of TCU from eleven studies [1,3,21,8,12,14–19] was 40.98% (95% CI=25.04-56.92; I^2^=99.61, P=0.001) **(Figure 2).**

## Subgroup analysis

Subgroup analysis was performed by using the country name Ethiopia and others, study setting, and sample size category. However, there was no effect on reducing the heterogeneity of the heterogeneity (I^2^). According to the seven studies of pooled effects in Ethiopia, the incidence of TCU was 47.19 (26.28-68.10), and according to four studies in other African countries, the incidence of TCU was 30.03 (18.11-41.94). After subgroup analysis by study setting, five studies used a community-based cross-sectional study design, for which the pooled prevalence was 55.56% (32.93-78.20). The remaining six studies used a health facility-based cross-sectional study design and had a pooled prevalence of 28.73% (19.67-37.78)**.** The pooled prevalence of TCU in studies that used health facilities as a source of data was lower than that in studies obtaining data from the community. This implies that nearly half of the children would not seek medical care after they had received TCU. According to our subgroup analysis by sample size, the pooled incidence in three studies with a sample size less than 500 was 25.30% (13.97-36.63).

The pooled incidence was 47.65% (26.04-69.25) for studies with sample sizes greater than 500 in seven studies (**Table 3**).

**Table 3.** Subgroup analysis for TCU in Africa

| **Sub-group** | **Author and year** | **Pooled OR (95% CI)** | **I^2^(P value)** |
| --- | --- | --- | --- |
| By Ethiopia | (Gebrekirstos et al., 2014) (Mitke, 2010)  (Kebede *et al.*, 2017) (Yirdaw et al., 2022) (Gebrekirstos et al., 2014)  (Bayih et al., 2020) | 47.19(26.28-68.10) | 99.64%(0.00) |
| By Other African countries | (Farouk et al., 2023) (Kambale *et al.*, 2018) (Adoga, 2011b) (Oluwatosin *et al.*, 2016)  (Djakounda, 1994) | 30.03(18.11-4194) | 97.96%(0.00) |
| By community based | (Kebede *et al.*, 2017) (Yirdaw et al., 2022) (Gebrekirstos et al., 2014) (Mitke, 2010) | 55.56(32.93-78.20) | 99.62%(0.00) |
| By Health facilities | (Farouk et al., 2023) (Kambale *et al.*, 2018) (Adoga, 2011b) (Oluwatosin *et al.*, 2016) (Djakounda, 1994) (Bayih et al., 2020) (Kefelew et al., 2023) | 28.73(19.67-37.78) | 97.45%(0.00) |
| Sample size >500 | (Bayih et al., 2020) (Kebede *et al.*, 2017)  Kefelew et al., 2023) | 25.30(13.97-36.63) | 95.84%(0.02) |
| Sample size <500 | (Farouk et al., 2023) (Kambale *et al.*, 2018) (Adoga, 2011b) (Djakounda, 1994) (Gebrekirstos et al., 2014) (Mitke, 2010)  (Yirdaw et al., 2022) (Gebrekirstos et al., 2014) | 47.65(26.04-69.25) | 99.72%(0.00) |

# Predictors of TCU

Based on this review, the predictors of traditional childhood uvulectomy according to pooled data from two studies were identified as rural residents and had a previous history of traditional uvulectomy. By pooling studies, rural resident mothers were 2.45 times more likely to undergo traditional uvulectomy than were those living in urban areas[16,18] (AOR=2.45; 95% CI=1.59- 3.32) (**Figure 3**).

Mothers with a history of previous traditional childhood uvulectomy were 8 times more likely to practice than were those with no history of uvulectomy (AOR=8.44; 95% CI=6.27-10.61) [1,3] (**Figure 4**).

In a single study by Yirdaw et al., mothers with a lack of information and who were perceived as uvula causes were almost 3 times and nearly 5 times more likely to practice, respectively, than were those informed about complications caused by TCU (AOR=2.98; 95% CI=2.40-3.56) and those informed about complications caused by TCU (AOR=4.89; 95% CI=4.64-5.14), respectively[1]. According to maternal attitudes, nearly fourfold more patients with uvulitis (inflammation and swelling of the uvula) not cured by medical treatment than did their counterparts (AOR=3.92; 955CI=3.28-4.56)[1].

According to other studies by Bayih et al., children who were home-delivered and male neonates were 6 and 4.56 times more likely to practice TCU, respectively, than were their counterparts (AOR=6.02; 95% CI=4.36-7.68) and at (AOR=4.56; 95% CI=3.08-6.06), respectively)[3].

According to studies by Kebede et al. and Kefelew et al., housewife mothers and those with unfavourable attitudes toward modern medical care were nearly three and four times more likely to practice TCU, respectively, than were their counterparts and positive attitudes toward health care (AOR=2.98; 95% CI=1.72-4.20) and AOR=4.31; 95% CI=2.17-6[16,18]/>, respectively).

# Discussion

This systematic review and meta-analysis aimed to assess the pooled incidence of TCU and its predictors in Africa. The pooled incidence of TCU was 40.98% (25.04-56.92). Even though the traditional medicines used were not described in this review, the most common utilizers of traditional medicine were found in India, China, and Africa. This might be due to traditional

medicine being used by the majority of the population and the dominant system of the health care delivery system in Africa. It was also part of their age-long cultural practices in the long run before modern medicine came in to place in many African countries[5,23].

This meta-analysis revealed that rural residents were 3 times more likely to practice TCU than were urban residents[16,18]. This might be because socioeconomic status plays a role in choosing traditional medicine in the community over modern health care[24]. From a modern health-seeking perspective, rural residents might have reduced willingness and poor infrastructure to access health care compared to urban residents, so they prefer to use traditional healers found nearby in their village[25–28]. Health-seeking behaviors in rural communities might also hinder parents’ choice of care during childhood illness [24,29–31].

Having a previous history of TCU was associated with eightfold greater odds of practising TCU than not having a history of TCU[1,3]. This finding could explain why experienced users of traditional medicine might be promoters in the community as evidence of the 'safety' of this procedure for those new utilizers. Traditional medicine is acceptable to the vast majority of people in developing nations due to the perceived inefficacy of modern medicine, the perceived incurability of disease with modern health care, beliefs about the effectiveness of traditional medicine, affordability, and feelings of embarrassment toward the modern care system[29,32,33].

# Limitations of the study

# Conclusion And Recommendations

Traditional childhood uvulectomy is quite common in Africa. The pooled prevalence estimate of TCU from eleven studies was nearly 41%. Mothers who lived in rural areas and had a history of previous practices related to TCU had greater odds of practicing TCU than did their counterparts. Thus, studies focused on the burden of the problem of childhood admissions caused by TCU are recommended to be more beneficial. Moreover, it was advised to develop a project involving the problem in maternal-child health (MCH) packages.

## Availability of data

The data will be available upon request to corresponding author ([solomondemis@gmail.com](mailto:solomondemis@gmail.com)).

## Ethics approval

Not applicable.

## Conflicts of interest

All authors declared no conflicts of interests

## Author contributions

**Conceptualization:** Solomon D. Kebede

**Data curation:** Kindu Agmas

**Formal analysis and methodology:** Solomon D. Kebede **Supervision and commenting:** Demewoz Kefale and Amare Kassaw **Writing original draft:** Solomon D. Kebede and Tigabu M. Aytenew **Writing, review and editing:** Solomon D. Kebede

## Funding

No funding was received for this review.

## Patient and public involvement

Patient and/or the public were not involved in the design, or conduct, or reporting, or dissemination plans of this research.

**Abbreviations: AOR**-Adjusted Odds Ratio, **CI**-Confidence Interval, **DRC**-Democratic Republic of Congo, **MCH**-Maternal Child Health, **TCU**-Traditional Childhood Uvulectomy, **WHO-**World Health Organization.

# References

1. Region A. Practice and associated factors of traditional uvulectomy among caregivers hafile:///C:/Users/HAB-TECH/Desktop/ajol-file- journals_302_articles_174662_submission_proof_174662-3601-447055-1-10-

20180717.pdfving children less than 5 years old in South Gondar . 2022;975: 1–17. doi:10.1371/journal.pone.0279362

2. Baker EJ, Sanei LC, Franklin N. Early initiation of and exclusive breastfeeding in large- scale community-based programmes in Bolivia and Madagascar. J Heal Popul Nutr. 2006;24: 530–539.
3. Alebachew Bayih W, Minuye Birhan B, Yeshambel Alemu A. The burden of traditional neonatal uvulectomy among admissions to neonatal intensive care units, North Central Ethiopia, 2019: A triangulated crossectional study. PLoS One. 2020;15: e0234866. doi:10.1371/journal.pone.0234866
4. Peter S, Kibira S, Namutundu J, Id JK, Kiwanuka N, Nankabirwa V, et al. PLOS GLOBAL PUBLIC HEALTH Uvula infections and traditional uvulectomy : Beliefs and practices in Luwero district , central Uganda. 2023; 1–14. doi:10.1371/journal.pgph.0002078
5. Che C-T, George V, Ijinu TP, Pushpangadan P, Andrae-Marobela K. Chapter 2 – Traditional Medicine. Pharmacognosy. 2017; 15–30.
6. Ijaz N. Pr ep rin t n ot pe er r ev iew Pr ep rin t n ot pe er ed. : 1–37.
7. Ndu IK, Duke-onyeabo CP, Onu N, Nnamani OG, Iheji CC. Traditional Uvulectomy: A common and potentially life-threatening practice in a developing country. 2022; 10–13.
8. R MK, Balibuno Y, N IF, J BK, G FM, Masumbuko B. Uvulectomie traditionnelle , une pratique courante au Sud-Kivu en République démocratique du Congo Traditional uvulectomy , a common practice in South Kivu in the Democratic Republic of Congo. 2018; 176–181.
9. M A, SB A. Traditional Uvulectomy Among the Neonates: Experience in a Nigerian Tertiary Health Institution. Borno Med J. 2016;13: 16–20.
10. Hunter L. Uvulectomy - The making of a ritual. South African Med J. 1995;85: 901–902.
11. Munn Z, Stone JC, Aromataris E, Klugar M, Sears K, Leonardi-Bee J, et al. Assessing the risk of bias of quantitative analytical studies: introducing the vision for critical appraisal

within JBI systematic reviews. JBI Evid Synth. 2023;21. Available: https://journals.lww.com/jbisrir/fulltext/2023/03000/assessing_the_risk_of_bias_of_quanti tative.2.aspx

12. Adoga AA. The Traditionally Amputated Uvula amongst Nigerians : Still an Ongoing Practice. 2011;2011. doi:10.5402/2011/704924
13. Faigel. H. C. (1966). Tonsillectomy — a bloody mess. Clinics of Paediatrics 5: 652-653. : 921.
14. Gebrekirstos K, Abebe M, Fantahun A. A cross sectional study on factors associated with harmful traditional practices among children less than 5 years in Axum town , north Ethiopia , 2013. 2014; 1–7.
15. Gebrekirstos K, Fantahun A, Buruh G. Magnitude and Reasons for Harmful Traditional Practices among Children Less Than 5 Years of Age in Axum Town , North Ethiopia , 2013. 2014;2014.
16. Kebede K, Mulate K, Chekole M, Redahegn H, Demelash A. Prevalence and Associated Factors to Uvula Cutting on Under Five Children in Amhara Region , Debre Birhan Town

, 2016 / 2017. 2017; 1–9.

17. Mitke YB. Bloody Traditional Procedures Performed During Infancy in the Oropharyngeal Area Among HIV + Children : Implication from the Perspective of Mother-to-Child Transmission of HIV. 2010; 1428–1436. doi:10.1007/s10461-010-9681- 4
18. Kefelew E, Yihun M, Dawite F, Kadi A, Tofik E, Abera A, et al. Magnitude of Uvulectomy and Associated Factors Among Caregivers Having Under Five Children in Arbaminch Town Public Health Facility , Gamo Zone ,. 2023;4.
19. Oluwatosin S, Ayodeji S, Ameh F, David O, Banjo O, Salman A. International Journal of Pediatric Otorhinolaryngology Pro fi le of pediatric traditional uvulectomy in North-West Nigeria : The need for caution and education *. Int J Pediatr Otorhinolaryngol. 2016;88: 194–198. doi:10.1016/j.ijporl.2016.07.011
20. Adoga AA. The Traditionally Amputated Uvula amongst Nigerians : Still an Ongoing

Practice. 2011;2011: 2009–2012. doi:10.5402/2011/704924

21. Djakounda M. TRADITIONAL UVULECTOMY IN NIGER : HEALTH PROBLEM ? 1994;39: 1077–1082.
22. Kano A. Nigerian Journal of Perinatal and Neonatal Care. 2023;3: 22–28.
23. ajol-file-journals_209_articles_67959_submission_proof_67959-2485-140140-1-10- 20110715.pdf.
24. Musinguzi G, Anthierens S, Nuwaha F, Van Geertruyden JP, Wanyenze RK, Bastiaens H. Factors Influencing Compliance and Health Seeking Behaviour for Hypertension in Mukono and Buikwe in Uganda: A Qualitative Study. Int J Hypertens. 2018;2018. doi:10.1155/2018/8307591
25. Musoke D, Boynton P, Butler C, Musoke MB. Health seeking behaviour and challenges in utilising health facilities in Wakiso district, Uganda. Afr Health Sci. 2014;14: 1046–1055. doi:10.4314/ahs.v14i4.36
26. Shaikh BT, Hatcher J. Health seeking behaviour and health service utilization in Pakistan: Challenging the policy makers. J Public Health (Bangkok). 2005;27: 49–54. doi:10.1093/pubmed/fdh207
27. ajol-file-journals_336_articles_58296_submission_proof_58296-4009-103363-1-10- 20100823.pdf.
28. Prosser T. Edith Cowan University. Grants Regist 2024. 2023; 470–472. doi:10.1057/978- 1-349-96073-6_427
29. Latunji OO, Akinyemi OO. Factors Influencing Health-Seeking Behaviour Among Civil Servants in Ibadan, Nigeria. Ann Ibadan Postgrad Med. 2018;16: 52–60. Available: http://www.ncbi.nlm.nih.gov/pubmed/30254559%[0Ahttp:](http://www.pubmedcentral.nih.gov/a)//[www.pubmedcentral.nih.gov/a](http://www.pubmedcentral.nih.gov/a) rticlerender.fcgi?artid=PMC6143883
30. Uche EO. European Journal of Social Sciences Studies FACTORS AFFECTING HEALTH SEEKING BEHAVIOUR AMONG RURAL DWELLERS IN NIGERIA AND ITS IMPLICATION ON. 2017; 74–86. doi:10.5281/zenodo.400695
31. Hoeven M Van Der, Kruger A, Greeff M, Central B. Differences in health care seeking behaviour between rural and urban communities in South Africa Research population Research measuring instrument. Int J Equity Health. 2012;11: 1–9. Available: <http://www.equityhealthj.com/content/11/1/31>
32. Legesse FM, Babanto AM. Factors Associated With the Use of Traditional Medicine in Wolaita Zone, Southern Ethiopia. SAGE Open. 2023;13: 1–10. doi:10.1177/21582440231153038
33. Kenu A, Kenu E, Bandoh DA, Aikins M. Factors that promote and sustain the use of traditional, complementary and integrative medicine services at LEKMA hospital, Ghana, 2017: an observational study. BMC Complement Med Ther. 2021;21: 1–10. doi:10.1186/s12906-020-03185-y

PRISMA flow diagram of study selection process	[Click here to access/download;Figure;Figure 1.TIF](https://www2.cloud.editorialmanager.com/pone/download.aspx?id=37359317&guid=327d1cd6-d901-45dd-84a0-2893baf20806&scheme=1)

Pooled prevalence of childhood uvulectomy in Africa	[Click here to access/download;Figure;Figure 2.TIF](https://www2.cloud.editorialmanager.com/pone/download.aspx?id=37359319&guid=9d93fd53-8e74-440a-9e26-fbad9cd5fe31&scheme=1)

Pooled predictors of maternal rural residency on TCU in Africa	[Click here to access/download;Figure;Figure 3.TIF](https://www2.cloud.editorialmanager.com/pone/download.aspx?id=37359321&guid=4e210d30-ac8e-48db-919a-1844f62f7c5f&scheme=1)

Pooled predictors of previous history of uvulectomy on TCU in Africa

[Click here to access/download;Figure;Figure 4.tif](https://www2.cloud.editorialmanager.com/pone/download.aspx?id=37359325&guid=745d49be-486f-45d3-95ea-444a76c58f96&scheme=1)

PRISMA checklist

### [Click here to access/download](https://www2.cloud.editorialmanager.com/pone/download.aspx?id=37369918&guid=2e31106a-b54d-4a4f-b672-0a99193079c7&scheme=1)

[**Supporting Information** Supplementary file 1.pdf](https://www2.cloud.editorialmanager.com/pone/download.aspx?id=37369918&guid=2e31106a-b54d-4a4f-b672-0a99193079c7&scheme=1)

Quality Assessment of the Included Studies Using the Joanna Briggs Institute (JBI) Quality Appraisal Criteria

### [Click here to access/download](https://www2.cloud.editorialmanager.com/pone/download.aspx?id=37369919&guid=6ec0d860-bc1c-43da-a70e-add71a1bef17&scheme=1)

[**Supporting Information** Supplementary file 2.pdf](https://www2.cloud.editorialmanager.com/pone/download.aspx?id=37369919&guid=6ec0d860-bc1c-43da-a70e-add71a1bef17&scheme=1)
