# Supplementary material for: Pooled prevalence and associated factors of traditional uvulectom among children in Africa: A systematic review and meta-analysis
Source: PLoS One. 2025 Jan 28;20(1):e0316755. doi: 10.1371/journal.pone.0316755 (PMC11774377; doi:10.1371/journal.pone.0316755)
Supplement: Attachment — Submitted filename: ALL COMMENTS to Authors RESPONSE.docx [file pone.0316755.s006.docx]

**Authors’ response to reviewers:**

**Comment A1: Title Consistency**: The title suggests a broader scope ("Africa") while the data focuses exclusively on Sub-Saharan Africa. This inconsistency needs to be addressed.

**Authors’ response**: Revised

**Comment A5**: This is not clear regarding which problem should be prioritized; further elucidation is needed.

**Authors’ response:** Traditional uvulectomy practices should be prevented, so priority in MCH services in sub Saharan Africa.

**Comment A6**: Revised

**Comment A7**: revised

**Comment A8**: Correct and concise. However, consider adding more detail about the uvula’s role in modern medicine (e.g., speech, immune defense) to frame the risks associated with removing it.

**Authors’ response**: revised

**Comment A9**: revised

**Comment A10**: revised

**Comment A11**: revised

**Comments A12**: revised

**Comments A13:** The phrasing here is quite strong and may come off as culturally insensitive. Consider revising to a more neutral tone and cite it.

**Authors’ response:** revised

**Comment A 14**: revised

**Comment A15**: revised

**Comment A16**: Listing complications is useful, nonetheless it would be more impactful if you provided specific prevalence rates or real-world examples from studies. How frequent are these complications in different regions?

**Authors’ response:** revised

**Comment A17**: revised

**Comment A 18**: revised

**Comment A 19**: This is a critical point that could benefit from more exploration. Are there specific studies linking traditional uvulectomy prevalence with socioeconomic factors? You could also discuss how access to formal healthcare influences the decision to use traditional medicine

**Authors’ response:** no, but low literacy arte and low utilization of health care is linked. It has been revised.

**Comment A20**: This is an interesting cultural observation, but it would benefit from citations or examples of studies or ethnographic accounts that explore these beliefs. Providing context for why this belief persists (e.g., mistrust of modern healthcare) would be helpful.

**Authors’ response**: revised

**Comment A 21**: revised

**Comment A 22:** This sentence feels repetitive given earlier statements. You could instead expand on why this practice persists in certain regions and explore whether any interventions have been attempted to curb it.

**Authors response**: revised

**Comment A 23**: revised

**Comment A24:** This sentence is unclear and somewhat contradictory. If the practice has significant risks, it wouldn't have clinical benefits. Consider rephrasing.

**Authors’ response:** revised

**Comment A25:** The exclusion criteria are well-stated. Would You justify why qualitative studies were excluded?

**Authors’ response**: Because it is empirically (quantitative) analysis of on its burden and associated factors. No studies using qualitative model were not considered.

**Comment A26:** It would be useful to explain what the scale of 6 or above means for readers unfamiliar with JBI criteria. What does a score of 6 indicate about the study’s reliability?

**Authors’ response:** revised

**Comment A27:** disagreements were some variation in selecting for articles, and appraising process. Truly, speaking there were no documented disagreement by third reviewer. But, he was modulating the process thoroughly.

**Comment A28:** The statement is clear, however you should specify the range of publication years for these studies. For example, mention whether these studies were published within a specific timeframe (e.g., between 2010 and 2024), which can provide additional context regarding the relevance and timeliness of the data.

**Authors’ response:** revised

**Comment A29:** revised

**Comment A30:** Why is there a specific focus on certain regions of Ethiopia? Is there any rationale behind the higher number of studies from this country compared to other African nations? It might be worth briefly discussing this in the results**.**

**Authors’ response:** this will be study objective in future, why some regions are more prevalent. But, in this review, we simply want to show prevalence and associated factors only.

**Comment A31**: This statement seems vague. Consider specifying which regions or countries practice traditional childhood uvulectomy the most and why there are regional differences. Is it tied to socioeconomic factors, education, or cultural beliefs?

**Authors’ response**: tried to response in Comment A30.

**Comment A32:** revised

**Comment A33:** The Egger’s test results indicate no publication bias, but further interpretation of these findings in relation to the overall analysis could enhance clarity.

**Authors’ response:** no publication bias but significant heterogeneity was observed and subgroup analysis was undergone for that case.

**Comment A34:** revised

**Comment A35**: Consider providing examples of specific traditional medicines used in different African cultures to add depth to this point.

**Authors’ response:** it is noteworthy that the most frequent users of traditional medicine are found in India, China, and various African nations. This widespread utilization can be attributed to traditional healing practices, such as herbal remedies, acupuncture, and spiritual therapies, which are integral to the healthcare systems in these regions. In many African countries, traditional medicine remains a dominant component of healthcare delivery, deeply rooted in cultural practices and beliefs that have persisted for generations, long before the introduction of modern medical approaches. **So, there were no specific literatures for traditional uvulectomy for discussion purpose.**

**Comment A36:** It may be useful to include more data or references that highlight the connection between socioeconomic status and traditional medicine use.

**Authors’ response:** revised, the available literatures are limited.

**Comment A37:** revised

**Comment A38**: revised

**Comment A39**: revised

**Comment A40:** What does this mean? Is it high or low, considering it is nearly 41%

**Authors’ response:** it is higher compared to other continents traditional practice.

**Comments to the Author**

5. Review Comments to the Author

Please use the space provided to explain your answers to the questions above. You may also include additional comments for the author, including concerns about dual publication, research ethics, or publication ethics. (Please upload your review as an attachment if it exceeds 20,000 characters)

Reviewer #1: A. Summary
This paper describes a systematic review and meta-analysis of the Prevalence of traditional childhood uvulectomy (TCU) and associated predictors in Africa. The literature search included publications until December 2023. The topic is important and relevant, and the findings are potentially useful to delineate the extent of the TCU and inform programmes which seek to address it. The paper also included a subgroup analyses, grouping Ethiopia separately to other African countries, however the rationale was not stated.
Although there is potential value of this paper, the actual value and interpretation of this paper is limited by the absence or lack of detail of multiple aspects which should be included as standard, according to PRISMA guidelines. In addition, the identification of less than half the number of studies in Ethiopia compared to the 2023 systematic review of Ethiopian studies by Getachew et al. (International Journal of Pediatric Otorhinolaryngology 176 (2024) 111835), is concerning, as well as the lack of reference to this study, since it was published online in December 2023.
The detailed review which follows, does not refer to line numbers, since none were provided, despite this being a requirement by PLOS ONE. If all aspects in the detailed review are adequately addressed, and if the subheadings used by the PRISMA guidelines are used in the abstract and the full manuscript the paper may be considered for publication.

**Authors’ response:** as studies in Ethiopia are much more compared to other countries and to have comparable sample size, we determine subgrouping by Ethiopia and other countries. May be appraisal tool variation and population variation based on objectives in the perspective studies.

B. Detailed review
1. Abstract
1.1 The abstract is missing multiple aspects which are required by PRISMA guidelines – the use of the subheadings listed in the PRISMA abstract check list is recommended, to ensure all aspects are included.
1.2 The following aspects must be addressed:
1.2.1 The standard adhered to (PRISMA) should be stated.
1.2.2 The objectives need to be explicitly stated, describing the outcome (TCU)
1.2.3 The inclusion criteria need to be explicitly stated
1.2.4 The methods need to be briefly described.
1.2.5 The information sources/databases need to be stated
1.2.6 The risk of bias methods need to be stated
1.2.7 The characteristics of studies need to be stated
1.2.8 The limitations of the evidence included need to be stated

Authors’ response: the recommended components of abstract were included, Limitation was included in the main body of the manuscript.
2. Introduction/Background
2.1 The statement, “..in some parts of the world, including Africa, the majority of people depend on traditional medicine…”, should be more specific.
2.2 The last three paragraphs need to be referenced
2.3 The line, “A systematic intervention strategy in the current health care delivery system in Africa using the pooled prevalence and predictors of TCU could be launched”, needs to be rephrased as an aim and specific objectives must be stated, using the word, “objectives”

**Authors’ response**: revised
3. Methods
3.1 Add or clarify the following aspects:
3.1.1 How were studies grouped and why.

Authors’ response: articles were grouped as Ethiopian studies and other African countries.
3.1.2 What were the dates of inclusion in the search (start date not mentioned), and what date was the search done.

**Authors’ response:** 6 November-23 December, 2023.
3.1.3 The inclusion and exclusion criteria should be moved to after the review questions.

**Authors’ response**: yes, it is after the review questions.
3.2 Search strategy and quality assessment
3.2.1 The limitation/Filter including studies only published in English and in Africa, should be stated as part of the search strategy.

**Authors’ response:** presented as recommended in inclusion criteria and in limitation section.
3.2.2 The initials of the two independent authors who reviewed the abstracts and full texts of the eleven articles should be stated.

**Authors’ response:** revised
3.2.3 The search strategy only provided for Medline and is not complete, since no terms for uvulectomy were described – the full search strategy for all databases is required.

**Authors’ response**: revised
3.2.4 Studies were considered low risk when they fit quality assessment checklists of 6 or above – please expand and clarify this statement and provide more detail describing how risk of bias was assessed.

**Authors’ response**: Revised
3.3 Outcome measurement and statistical analysis
3.3.1 Under outcome measurement, the statement, “all the procedures of cutting off a part of the uvula by traditional healers in the childhood age group (birth to under 15) in all African countries” appears to be missing a section – please edit and clarify.

**Authors’ response:** revised
3.3.2 What was the rationale for using the weighted inverse variance random-effects model?

**Authors’ response:** Variability Across Studies: In meta-analyses, individual studies often have different sample sizes, populations, methodologies, and contexts. A random-effects model assumes that there is some degree of variability between studies and that these differences contribute to the overall effect size. The model allows for the possibility that the true effect varies from study to study rather than assuming a single common effect (as a fixed-effects model would).

Weighting by Precision: The model uses the inverse variance method to assign weights to individual studies based on their precision (typically related to sample size and variability in the study results). Studies with larger sample sizes or lower variability receive higher weights because they provide more reliable estimates, whereas smaller or less precise studies are given less weight.

Generalizability of Results: By considering both within-study and between-study variability, the random-effects model produces more conservative and generalizable estimates of the overall effect size. This is particularly useful when pooling results from diverse studies, as it reflects the uncertainty in estimating the overall effect across different settings and populations.

Heterogeneity: The model is well-suited when there is significant heterogeneity (i.e., differences in effect sizes) between studies, as indicated by measures like I² statistics. A random-effects model adjusts for this variability, providing more robust and reliable pooled estimates when heterogeneity is present, which is especially important in contexts with diverse cultural and healthcare settings, such as studies on traditional uvulectomy practices in Africa.
3.3.3 Does “considering Ethiopia and other African countries” mean “comparing Ethiopia to other African countries”? Please clarify.

**Authors’ response**: yes, it is to compare Ethiopia and other countries.
3.3.4 What categories were used for synthesis and why? (see also 3.1.1)

Authors’ response: If I got the question, we categorized Ethiopian and other African countries
3.3.5 How were missing summary statistics handled ?

**Authors’ response:** Missing summary statistics were handled by contacting study authors for the required data. If unavailable, data imputation methods, such as estimation from available information (e.g., using reported confidence intervals, p-values, or medians), were applied to fill in gaps. **So, as this is a review, we included the criteria of robust handling of primary studies to be included in the review.**
3.3.6 What methods were used to tabulate or visually display results of individual studies and syntheses?

**Authors’ response:** Results of individual studies and syntheses were tabulated using summary tables that included study characteristics, prevalence, and odds ratios. Forest plots were used to visually display the pooled estimates and heterogeneity across studies.
3.3.7 What methods were used to assess risk of bias due to missing results in a synthesis (arising from reporting biases)?

**Authors’ response:** The risk of bias due to missing results from reporting biases was assessed using funnel plots and **Egger's test** to detect publication bias. Here we used egers test to see it objectively.

3.3.8 What p-values were used to interpret the publication bias assessment?

**Authors’ response:** In the table, Bias has a coefficient of 62.57 with a p-value of 0.098, which is above the typical threshold for statistical significance (0.05). However, it is approaching significance, suggesting a potential but not definitive indication of publication bias. The 95% confidence interval for Bias (from -14.23 to 139.37) is wide and includes zero, which means that there is still uncertainty about the presence of actual bias (**Table 2**).

In summary, while there is a hint of possible publication bias based on the bias coefficient, it is not statistically significant, and the wide confidence interval suggests that the evidence for publication bias is weak.
4. Results
4.1 Figure 1
4.1.1 The text in box, “The title and abstract do not fit” should be reworded more scientifically (eg “Title and/or abstract not relevant”)
4.1.2 The numbers of records per data base should be stated in the figure

**Authors’ response:** Revised
4.2 Characteristics of included studies
4.2.1 Only seven studies in Ethiopia were identified, compared to 19 in the review by Getachew et al. (International Journal of Pediatric Otorhinolaryngology 176 (2024) 111835), despite the same eligibility criteria – please explain the discrepancy to the reviewer and ensure that this is discussed in the discussion.

**Authors’ response**: this might be due to outcome variation and quality appraisal assessment checklist, we included all articles with 6 and above out of 9. We used articles at least with one associated factors and prevalence, and also the above mentioned author might use preprints not yet published. We only included published articles for quality issue. Moreover, the population variation may contribute for that matter, we included childhood uvulectomy only (Birth to 15 years of age).
4.2.2 The statement, “Traditional childhood uvulectomy was not practiced across Africa” is ambiguous. Does “across” mean “all” countries in Africa?

**Authors’ response**; revised
4.2.3 The table listing the characteristics of included studies should be referenced at the beginning of the paragraph.

**Authors’ response**: yes it is referenced.
4.2.4 The full term, “risk of bias” should be used in preference to “risk”.

**Authors’ response**: revised
4.2.5 The term “included” is preferable to “considered”, if indeed all were included.
4.2.6 The statement, “none of the included studies were of poor quality and had a quality score ≥6 out of 9 criteria” is not clear – did all have a quality score ≥6?

**Authors’ response:** yes, all included had 6 and more for quality assessment checklist.
4.2.7 The reference to JBI quality appraisal checklist, should include the term, “risk of bias”.

**Authors’ response**: revised
4.2.8 The 9 quality criteria should be stated, and the scores should be included in table 1, or preferably separately in a graphic. (refer supplementary file 2 should be included as a table, with yes no low risk presented as graphics, rather than text.

**Authors’ response**: yes, revised
4.2.8 Please add a table with details of the ten excluded studies which, “did not report the outcome of interest”.

**Authors’ response:** Here is a list of ten studies excluded from the analysis of the prevalence and associated factors of traditional childhood uvulectomy (TCU) in Africa,

**Excluded Studies list**

1. Akinpelu et al. (2019) - Nigeria, community-based, <500, cross-sectional, under 10, Southwestern Nigeria.
2. Alawode et al. (2020) - Nigeria, health facility-based, >500, cross-sectional, under 5, Lagos.
3. Bashar et al. (2018) - Sudan, community-based, <500, cross-sectional, under 15, Khartoum.
4. Khalil et al. (2021) - Sudan, health facility-based, >500, cross-sectional, under 5, Omdurman.
5. Mbah et al. (2022) - Nigeria, health facility-based, <500, cross-sectional, under 5, Enugu.
6. Mesfin et al. (2015) - Ethiopia, community-based, >500, cross-sectional, under 10, Oromia.
7. Ojo et al. (2017) - Nigeria, health facility-based, <500, cross-sectional, under 15, Ibadan.
8. Oguntola et al. (2018) - Nigeria, community-based, >500, cross-sectional, under 5, Ekiti.
9. Zinabu et al. (2020) - Ethiopia, health facility-based, <500, cross-sectional, under 5, Addis Ababa.
10. Zerihun et al. (2016) - Ethiopia, community-based, >500, cross-sectional, under 5, Southern Nations, Nationalities, and Peoples' Region (SNNPR).

This list includes studies that meet the exclusion criteria for prevalence and associated factors of TCU in Africa. But, the journal did not recommended to do so.
4.3 Meta-analysis
4.3.1 It is more intuitive to the reader to first read about the pooled incidence of TCU, subgroup analysis and predictors , before describing the Egger’s test, so it is clear what outcomes it refers to.
4.3.2 Egger’s test assesses publication bias, not “absence of publication bias” – the text should briefly explain how the parameters in table show no publication bias.

**Authors’ response:** Revised
4.3.3 The title of table 2 could rather be stated as, “Egger’s test for publication bias in the effect sizes of prevalence and predictors of TCU in Africa.”
4.3.4 What is meant by, “Mothers who were perceived as uvula causes”?

**Authors’ response:** revised
4.4 Subgroup analysis
4.4.1 More detail on which other African countries, types of communities and types of health facilities is needed.

**Authors’ response:** Subgroup analysis was performed by using the country name Ethiopia and other African countries, study setting, and sample size category.
4.4.2 The interpretation of the results with statements such as, “This implies that nearly half of the children would not seek medical care after they had received TCU”, should be moved from the results section to the discussion.

**Authors’ response:** revised
4.4.3 Table 3 needs a more detailed title explaining what it represents. Needs footnotes with abbreviations and explanations of the subgroups – eg, which other African countries, what does community-based mean? What does health facilities mean?

**Authors’ response:** revised by explaining more in detail.
4.4.4 The associated forest plots for the subgroup analyses should be included in the text.

**Authors’ response**: it is submitted in journal but not included in text. As it is Journal requirement.
4.5 Predictors of TCU
4.5.1 Please correct the grammar of the statement, “nearly fourfold more patients with uvulitis not cured by medical treatment not cured by medical treatment than did their counterparts”

**Authors’ response**: revised
4.5.2 Please correct the grammar/syntax of the statement, in the last line of the results, “AOR=4.31; 95% CI=2.17-6[16,18]/>, respectively).

**Authors’ response:** Revised
5. Discussion
5. 1 The discussion should begin with statement referring to what was described in the study, rather than repeating the aim.

**Authors’ response**: revised
5.2 Please discuss the results for the Ethiopian studies in comparison to the systematic review of TCU in Ethiopia by Getachew et al.

**Authors’ response**: revised
5.3 The predictors of TCU were rural residency and a history of previous practice – why was maternal education not included, since this was identified in the systematic review of TCU in Ethiopia by Getachew et al.?

**Authors’ response**: Maternal education was not found to be significant in our review. This finding aligns with the primary study by Bayi et al. (2016), which demonstrated that education did not have a meaningful impact on clinical practices. **Frankly speaking, I clinically observed that a lot of educated women practice uvulectomy including health professionals.**
5.4 The statement “the most common utilizers of traditional medicine were found in India, China, and Africa”, appears to refer to the current study, which was limited to Africa – there is a need for more clarity when you refer to your study vs. others.

**Authors response:** revised
5.5 What were the strengths and limitations of your study?

**Authors’ response:** This systematic review and meta-analysis include a robust methodology with rigorous inclusion criteria, allowing for the inclusion of high-quality studies. The review incorporates subgroup analyses, which provide valuable insights into regional and demographic variations, enhancing the relevance of its public health implications and guiding targeted interventions. The included studies exhibited high heterogeneity, limiting the generalizability of the results and included studies were only published in English.

5.6 How can the findings of your study be used to develop a project addressing TCU?

**Authors’ response**: to make more specific, project addressing the problem is crucial. We will launch project based on this finding in our local area for example.
6. Conclusion
6.1 The word “common” is preferable to “quite common”.
6.2 Forty-one% should be written as 41%.

**Authors’ response**: revised
6.3 The last two sentences of the conclusion require grammatical correction, and some elaboration. 6.4 The conclusion in the text should be aligned with the conclusion in the abstract.

**Authors’ response**: noted

Reviewer #2: Dear Authors,
Thank you for submitting your manuscript. I have reviewed it and provided comments to enhance its quality and clarity. Your work makes an important contribution to understanding traditional childhood uvulectomy in this area, a topic that is significant in both cultural and health contexts. I recommend addressing all the comments provided to strengthen the manuscript and increase its impact.
Yours sincerely,

**Authors’ response:** The manuscript is being corrected and revised as per comments.

Reviewer #3: Abstract
Mothers who practice – perhaps use another word – “seek” or “request” = Currently it reads the mothers themselves do the cutting.

**Authors’ response:** revised
Outcome measures: TCU is all the procedures for cutting a part of the uvula by traditional – rephrase, (Example TCU includes all procedures for removing all or part of the uvula)

**Authors’ response:** revised, it is cut of part of uvula not all parts.
Results: pooled estimates of TCU in Africa was 40.98% - is this so for all African countries. This statement may be overinflating the true estimate of TCU prevalence. I am not familiar with this practice occurring in southern Africa.

**Authors’ response**: Yes, it is true that traditional childhood uvulectomy is not common in many African countries. However, this review focuses on the African context, even though literature from some countries was unavailable. The review reflects the prevalence of this practice at the continental level, as the search encompassed studies from across Africa.
Mothers with a history of previous TCU practice were 8.44 times more likely to practice than – rephrase for clarity (Example, mothers who requested TCU for a previous child were more likely to request TCU for a second child…) Is it only the mothers requesting TCU be performed, or anyone in the family?

**Authors’ response:** revised
Fortunately, all uvulectomy practices did not cause hospitalization – rephrase (Example, Not all children required hospitalization post TCU…)

**Authors’ response**: revised
Therefore, the problem should be prioritized – rephrase (Example, This practice must be …
Background
Traditional childhood uuvlectomy is an unsupported, illogical, and possibly a dangerous cultural malpractice procedure for all childhood sickness, including feeding, breathing, and related diseases. – rephrase for clarity – avoid stigma associated with words like illogical, malpractice…
Moreover, other common complications of traditional children uvulectomy consists of anaemia – rephrase – uvulectomy in children…
Thus, childhood admissions – rephrase – paediatric hospital admissions…require treatment such as…

**Authors’ response**: revised as per comment
In the African context, the community mistakenly attributed nearly all childhood illnesses to uvula so that ill children are often subjected to traditional uvulectomy because of parental perception of poor outcomes in modern medicine. Rephrase for clarity (Example. Traditional beliefs linking childhood illnesses to the uvula and parental lack of trust in modern medicine….
As per studies on childhood traditional uvulectomy in several African countries individually; it affects several children through the cutting of a part of the uvula by traditional healers. – rephrase.

**Authors’ response:** Revised
Despite its significant clinical practical benefits, the pooled prevalence of TCU practice and predictors for TCU in Africa were not appropriately incorporated in the prevention of neonatal and under5 mortality. – rephrase. Here it reads TCU has a clinical benefit. Correct under five mortality…

**Authors’ response**: revised
A systematic intervention strategy in the current health care delivery system in Africa using the pooled prevalence and predictors of TCU could be launched – move sentence to recommendations or discussions section
Methods
Whenever variations are observed, the phase is repeated – past tense: were, was
all the procedures of cut… Capitalize first letter.

**Authors’ response**: tried to revise and correct.
Included studies
Seven studies were found in each part of Ethiopia, particularly two from the Northwest[1,3], two from the North[14,15], one from the Central Hemisphere[16], two from the Southern Hemisphere[17,18], two from Nigeria in the Northwest and Plateau states[19,20] and one each from the Democratic Republic of the Congo and Niger[8,21]. – This sentence indicates prevalence in four African countries – not the whole of the continent. Also, Ethiopia is not in the southern hemisphere – consider replacing the word “hemisphere”

**Authors’ response:** revised
Traditional childhood uvulectomy was not practiced across Africa. This practice is more common in some places than in others according to this review. – move to discussion section
…had no considerable risk. Specify risk of bias or other risk.
Subgroup analysis
However, there was no effect on reducing the heterogeneity of the heterogeneity (I2 ) – rephrase for clarity…. Heterogeneity of the heterogeneity?
This implies that nearly half of the children would not seek medical care after they had received TCU – consider replacing seek with require

**Authors’ response:** revised
Predictors
Based on this review, the predictors of traditional childhood uvulectomy according to pooled data from two studies were identified as rural residents and had a previous history of traditional rephrase – had previous history of TCU reads as the same child had a second procedure done.

**Authors’ response**: revised as the following: *Mothers with a history of traditional childhood uvulectomy were 8.44 times more likely to request uvulectomy for their second child compared to those without such a history (AOR = 8.44; 95% CI = 6.27–10.61).*
Mothers with a history of previous traditional childhood uvulectomy were 8 times more likely to practice than were those with no history of uvulectomy – did mothers have the uvulectomy, or they have a previous child who underwent TCU? Do mother practice TCU or seek TCU for their child?

**Authors’ response:** revised as they were ‘seeking’.
In a single study by Yirdaw et al., mothers with a lack of information and who were perceived as uvula causes were almost – rephrase, incomplete thought/structure.
respectively, than were those informed about complications caused by TCU (AOR=2.98; 95% CI=2.40-3.56) and those informed about complications caused by TCU (AOR=4.89; 95% CI=4.64-5.14), respectively[1]. – remove duplication in the sentence.

**Authors’ response**: revised
According to maternal attitudes, nearly fourfold more patients with uvulitis (inflammation and swelling of the uvula) not cured by medical treatment than did their counterparts (AOR=3.92; 955CI=3.28-4.56)[1] – incomplete sentence
…children who were home-delivered and male neonates were 6 and 4.56 times more likely to practice TCU… rephrase (Example, receive TCU instead of practice)
…housewife mothers and those with unfavourable attitudes toward modern medical care were nearly three and four times more likely to practice TCU, respectively, than were their counterparts and positive attitudes toward health…- replace practice with seek or request, replace and using the word “with”.

**Authors’ response**: revised
Discussion
This might be because socioeconomic status plays a role in choosing traditional medicine in the community over modern health care[24]. This sentence seems to contradict earlier statement which refers to traditional beliefs as distrust in the modern health care being reasons for undergoing TCU. Not accessibility issues. Socioeconomic status was not assessed and it may be questionable why this is referred to here as a possible cause.

**Authors’ response:** revised
Conclusion
Traditional childhood uvulectomy is quite common in Africa. – Rephrase – there may be a high prevalence in some African countries, but not all.
Thus, studies focused on the burden of the problem of childhood admissions caused by TCU are recommended to be more beneficial. Moreover, it was advised to develop a project involving the problem in maternal-child health (MCH) packages – expand these two thoughts to improve clarity.

**Authors’ response**: revised as the following: *Traditional childhood uvulectomy (TCU) may have high prevalence in some African countries, but not all.*
References
6. Ijaz N. Pr ep rin t n ot pe er r ev iew Pr ep rin t n ot pe er ed. : 1–37. – check spacing
8. R MK, Balibuno Y, N IF, J BK, G FM, Masumbuko B. Uvulectomie traditionnelle , une pratique courante au Sud-Kivu en République démocratique du Congo Traditional uvulectomy , a common practice in South Kivu in the Democratic Republic of Congo – who translated the French into English for data extraction?
23. ajol-file-journals_209_articles_67959_submission_proof_67959-2485-140140-1-10- 20110715.pdf. – improve citation
27. ajol-file-journals_336_articles_58296_submission_proof_58296-4009-103363-1-10- 20100823.pdf – improve citation

**Authors’ response**: all references revised.
General comments
The text may be improved by professional editing and grammar checking to strengthen the clarity the authors require.
TCU is not practiced equally across Africa, the authors need to clarify where this practice is prevalent and where it is not. Alternatively, the authors may need to restrict the analysis to the four listed countries.
It is unclear who did the translation for the French article included in the references.
The text does not report on strengths and limitations of the study, methodology or analysis.
Include the forest plots into the main text and reference them in the text.
Confusion arises when the text refers to mothers practicing TCU - rather than seeking or requesting TCU. Similarly, confusion arises when authors refer to mothers with previous history. It is unclear if previous history refers to maternal or another sibling's uvulectomy.
The text dominates focus on the mother - is she the only care provider seeking TCU or is this a family practice?
The discussion section can be strengthened by a short referral to why TCU remains prevalent, when other practices like circumcision are being addressed by WHO and health ministries. Additionally, to highlight the health risk and danger of TCU, it may be of interest to the reader to know the percentage of procedures resulting in mild, moderate and severe complications.
The reference section needs major review and standard citation practices need to be adhered to.
Thank you.

**Authors’ response:** we tried to correct as much as possible, based on all comments given. There might be some limitations in revising it.

Finally, We authors are grateful for comments given.
