# Supplementary material for: Pooled prevalence and associated factors of traditional uvulectom among children in Africa: A systematic review and meta-analysis
Source: PLoS One. 2025 Jan 28;20(1):e0316755. doi: 10.1371/journal.pone.0316755 (PMC11774377; doi:10.1371/journal.pone.0316755)
Supplement: Attachment — Submitted filename: Response to Reviewers comment(R3).docx [file pone.0316755.s008.docx]

Journal Requirements:
Please review your reference list to ensure that it is complete and correct. If you have cited papers that have been retracted, please include the rationale for doing so in the manuscript text, or remove these references and replace them with relevant current references. Any changes to the reference list should be mentioned in the rebuttal letter that accompanies your revised manuscript. If you need to cite a retracted article, indicate the article’s retracted status in the References list and also include a citation and full reference for the retraction notice.
Reviewers' comments:
Reviewer's Responses to Questions

**Comments to the Author**
1. If the authors have adequately addressed your comments raised in a previous round of review and you feel that this manuscript is now acceptable for publication, you may indicate that here to by pass the “Comments to the Author” section, enter your conflict of interest statement in the “Confidential to Editor” section, and submit your "Accept" recommendation.

Reviewer #3: (No Response)

2. Is the manuscript technically sound, and do the data support the conclusions?
The manuscript must describe a technically sound piece of scientific research with data that supports the conclusions. Experiments must have been conducted rigorously, with appropriate controls, replication, and sample sizes. The conclusions must be drawn appropriately based on the data presented.

Reviewer #3: Partly

3. Has the statistical analysis been performed appropriately and rigorously?

Reviewer #3: Yes

4. Have the authors made all data underlying the findings in their manuscript fully available?
The [PLOS Data policy](http://track.editorialmanager.com/CL0/http:%2F%2Fwww.plosone.org%2Fstatic%2Fpolicies.action%23sharing/1/010f01937836915f-cfb5b8fe-787c-4cce-9f32-f88603c9d212-000000/FnQgOs_wAiGcmiRlWDqe5ocJE3uIna3nZTPVOW0-xPQ=186) requires authors to make all data underlying the findings described in their manuscript fully available without restriction, with rare exception (please refer to the Data Availability Statement in the manuscript PDF file). The data should be provided as part of the manuscript or its supporting information, or deposited to a public repository. For example, in addition to summary statistics, the data points behind means, medians and variance measures should be available. If there are restrictions on publicly sharing data—e.g. participant privacy or use of data from a third party—those must be specified.

Reviewer #3: Yes

5. Is the manuscript presented in an intelligible fashion and written in standard English?
PLOS ONE does not copyedit accepted manuscripts, so the language in submitted articles must be clear, correct, and unambiguous. Any typographical or grammatical errors should be corrected at revision, so please note any specific errors here.

Reviewer #3: Yes

6. Review Comments to the Author
Please use the space provided to explain your answers to the questions above. You may also include additional comments for the author, including concerns about dual publication, research ethics, or publication ethics. (Please upload your review as an attachment if it exceeds 20,000 characters)

Reviewer #3: Congratulations. The grammar, sentence structure and flow of the text has greatly improved and thus provides clearer insight. Minor comments and suggestions are attached.
My major concern is the lack of clarity (perhaps on my part) about the agents involved in the practice of TCU. To my understanding, there are three agents in this transaction.
1. The traditional healer – the one who performs the procedure and who practices TCU
2. The mother/parent – the one who seeks/requests this procedure for their child
3. The child who receives or undergoes this procedure (I assume not by choice)
The language in the text implies mothers practice and undergo this procedure. The role of the three agents outlined above are not clearly delineated in the text and confuse me as to who is responsible for doing the cutting (practice) and who had what done to them.
Are the authors saying that mothers who themselves had TCU done to them when they were children are more likely to have their own children undergo TCU? Or are the authors saying that mothers who have older children, who have undergone TCU with or without complications) are more likely to seek TCU for their younger children? The difference is subtle but important for interpretation of ongoing use and provision of traditional health care services in general.

**Authors’ response:** I sincerely appreciate the comments and acknowledge their importance. They raise very crucial points.

To clarify, we are referring to mothers whose children underwent a traditional uvulectomy. The mothers sought out traditional healers to perform the procedure on their children, but the mothers themselves did not perform it.

Regarding the reference to a previous history of uvulectomy, this indicates mothers who have one or more children who have already undergone the procedure.

Thank you for bringing up these points, as they help refine the understanding and presentation of the context.

Comments/Suggestions below:
Line number
Abstract:
29 ...associated of TCU… – there is a word missing
47 “perform”- suggest “seek” or “request”
52 …in Africa… – over inflating of results. Suggest to amend to read “…in some African countries…”

**Authors’ response**: revised as per comments.

Text
71 double period
88 incomplete sentence – suggest, remove the word “as”
90 unsuportedand – add space; remove the word “possibly” as you are confirming the danger of this practice later in the paragraph.
95 …during the same session… - suggest replace “session” with “procedure” – adds clarity.
135, 138, 139 Change oorder of words to align with P E C O (Context before outcome)
143 & 165 associated factors or “predictors”?

**Authors’ response**: revised as per comments.

187 & 188 use past tense (were,was instead of are, is)
212 Did Science Direct yield a zero result search? This may be worth mentioning.
Page 12 restarts line numbers as 1.

**Authors’ response**: Yes, there were no studies included for analysis from ScienceDirect, but there were five excluded articles at screening stage found in the search. At that stage, I considered only the databases that contributed to the final articles analyzed in this review.
1 risk of what? Specify if this relates to risk of bias.

**Authors’ response:** Risk of bias.
63 predictors of TCU …as rural residents and had a previous history to TCU… - sentence is slightly confusing – I am still unclear if “previous history” refers to the mother who herself has undergone the procedure of TCU or if she has had a previous child whom underwent the procedure. This aspect needs clarity!

**Authors’ response:** revised as follows:

‘’Mothers residing in rural areas were 2.45 times more likely to have a child experienced TCU compared to those in urban areas’’

‘’Mothers with a history of having a previous child who experienced TCU were 8.44 times more likely to seek the procedure for their other children compared to mothers without such a history.’

64 …mothers were more likely to undergo… - again, this implies the women themselves are having the uvulas removed – and not their children.

**Authors’ response:** revised as: *By pooling studies, rural resident mothers were 2.45 times more likely to have a child who underwent traditional childhood uvulectomy (TCU) compared to mothers living in urban areas.*
68 &73 &76 & 86 as with line 63… -did the mothers themselves have the TCU or did they seek this for an older child. Also, Mothers do not “practice” this procedure – they either seek or request this for their children… Please clarify this across the document.

**Authors’ response**: Revised
78 patients with uvulitis – is this in the adult population or the paediatric population – this is unclear.
83 children practice – children cannot do this procedure themselves (practice), they can undergo or receive this procedure.

**Authors’ response:** revised as ‘*According to maternal attitudes, children with uvulitis (inflammation and swelling of the uvula) that did not respond to medical treatment were nearly four times more likely to undergo traditional uvulectomy compared to their counterparts’*.
