# Supplementary material for: Pooled prevalence and associated factors of traditional uvulectom among children in Africa: A systematic review and meta-analysis
Source: PLoS One. 2025 Jan 28;20(1):e0316755. doi: 10.1371/journal.pone.0316755 (PMC11774377; doi:10.1371/journal.pone.0316755)
Supplement: Attachment — Submitted filename: Response to Reviewers comment(R4).docx [file pone.0316755.s009.docx]

We sincerely appreciate the reviewers’ valuable comments and acknowledge their importance in strengthening our manuscript. Below are our responses to the specific points raised:

**Reviewer #3: Minor edits**

1. **Line 188**: Change 'are' to 'were.'
   **Authors’ response**: The correction has been made by replacing 'are' with 'were.'
2. **Line 62**: "...mothers with older children who had a previous history..."
   **Authors’ response**: This section has been revised as per the comment for improved accuracy.
3. **Line 67**: Change 'underwent' to 'undergo.'
   **Authors’ response**: The change has been implemented, and 'underwent' has been replaced with 'undergo.'
4. **Line 83**: Replace 'to have a child with TCU' with 'to receive TCU.'
   **Authors’ response**: The sentence has been revised as suggested.
